# The abscisic acid–responsive element binding factors *MAPKKK18* module regulates abscisic acid–induced leaf senescence in *Arabidopsis*

**DOI:** 10.1016/j.jbc.2023.103060

**Published:** 2023-02-24

**Authors:** Guoying Zhao, Qian Cheng, Yuting Zhao, Feifei Wu, Bangbang Mu, Jiping Gao, Liu Yang, Jingli Yan, Hanfeng Zhang, Xing Cui, Qinqin Chen, Fangxiao Lu, Qianqian Ao, Asma Amdouni, Yuan-Qing Jiang, Bo Yang

**Affiliations:** 1 State Key Laboratory of Crop Stress Biology for Arid Areas, College of Life Sciences, Northwest A & F University, Yangling, Shaanxi, China; 2 National Key Laboratory of Plant Molecular Genetics, Shanghai Institute of Plant Physiology and Ecology, Chinese Academic of Sciences, Shanghai, China

**Keywords:** ABA-responsive element binding factors (ABFs), abscisic acid (ABA), leaf senescence, MAPKKK18, transcriptional regulation

## Abstract

The mitogen-activated protein kinase kinase kinase 18 (MAPKKK18) has been reported to play a role in abiotic stress priming in long-term abscisic acid (ABA) response including drought tolerance and leaf senescence. However, the upstream transcriptional regulators of *MAPKKK18* remain to be determined. Here, we report ABA-responsive element binding factors (ABFs) as upstream transcription factors of *MAPKKK18* expression. Mutants of *abf2*, *abf3*, *abf4*, and *abf2abf3abf4* dramatically reduced the transcription of *MAPKKK18*. Our electrophoresis mobility shift assay and dual-luciferase reporter assay demonstrated that ABF2, ABF3, and ABF4 bound to ABA-responsive element *cis*-elements within the promoter of *MAPKKK18* to transactivate its expression. Furthermore, enrichments of the promoter region of *MAPKKK18* by ABF2, ABF3, and ABF4 were confirmed by *in vivo* chromatin immunoprecipitation coupled with quantitative PCR. In addition, we found that mutants of *mapkkk18* exhibited obvious delayed leaf senescence. Moreover, a genetic study showed that overexpression of *ABF2*, *ABF3*, and *ABF4* in the background of *mapkkk18* mostly phenocopied the stay-green phenotype of *mapkkk18* and, expression levels of five target genes of ABFs, that is, *NYE1*, *NYE2*, *NYC1*, *PAO*, and *SAG29*, were attenuated as a result of *MAPKKK18* mutation. These findings demonstrate that ABF2, ABF3, and ABF4 act as transcription regulators of *MAPKKK18* and also suggest that, at least in part, ABA acts in priming leaf senescence *via* ABF-induced expression of *MAPKKK18*.

Leaf senescence is the final stage duuring leaf development, which precedes cell death (1). This process is necessary for plants’ fitness because the degeneration of chloroplasts, mitochondria, and nuclei allows for remobilizing nutrients and metabolites from old organs to young leaves and developing fruits (2). Its proceeding is basically regulated by the developmental age. Moreover, leaf senescence is also affected by a variety of internal and external signals that are integrated into developmental ages (2). Major phytohormones, including abscisic acid (ABA), jasmonic acid, ethylene, and salicylic acid; brassinosteroids; cytokinins; gibberellic acid; and auxin have all been documented to positively or negatively modulate leaf senescence (3).

ABA functions as one of the most effective phytohormones to promote leaf senescence. Exogenously applied ABA promotes leaf senescence (4), induces the expression of many senescence-associated genes (SAGs) (5) and promotes chlorophyll degradation (6, 7, 8, 9). The endogenous ABA level increases during the progression of leaf senescence in many plants (10). Moreover, elevated ABA levels and activated ABA signaling through a variety of abiotic and biotic stresses result in senescence (11).

The ABA core signaling is composed of receptor proteins Pyrabactin Resistance 1 (PYR1) and PYR1-Like (PYL) (12, 13), clade A Protein Phosphatase type 2C (PP2Cs) (12, 13, 14), Sucrose nonfermenting Related Protein Kinase 2s (SnRK2s) (15, 16), ABA-Responsive Element (ABRE) Binding Factors (ABFs) (17, 18, 19) and downstream Late Embryogenesis-Abundant (LEA)-like ABA-responsive genes (20). Once PYR/PYLs bind ABA (12, 13), the complex prevents PP2Cs from dephosphorylating SnRK2s (21, 22). Activated SnRK2s then transphosphorylate ABFs to activate the transcription of ABA-responsive LEA-like genes (18, 23, 24, 25). It has been reported that several components of ABA signaling are the causal factors of senescence (6, 9, 26, 27), including PYL9- SnRK2.6- ABF2 (9), ABF2/3/4 (6), Receptor-like Kinase 1 (RPK1) (27) and Highly ABA-Induced PP2C 1 (HAI1/SAG113) (26).

ABA signal transduction also cross talks with other signaling pathways. Among them, Mitogen-Activated Protein Kinase (MAPK/MPK) cascades are of particular interest as they are known for converting signals transduced by receptors into diverse cellular responses (28). MAPK modules consist of three titers of enzymes including MAPKKK, MKK, and MPK, which phosphorylate and thereby activate each other in a sequential way (28). As a result, activated MPKs then target and phosphorylate substrates to modulate their activity or stability and finally lead to specific responses to biotic and abiotic stresses, hormone stimuli, and developmental processes (29). MAPKKKs, with the largest number (80 in *Arabidopsis*) in the MAPK cascade, are rarely studied compared with MKKs and MPKs. So far, only a few MAPKKKs mediating ABA or relevant signalings have been demonstrated, including mitogen-activated protein kinase kinase kinase (MAPKKK) 18 (30, 31), MAPKKK20 in root elongation and MAPKKK16 in seed germination, cotyledon greening, and root growth (32, 33).

We previously showed that *MAPKKK18* from oilseed rape (*Brassica napus* L.) was induced by multiple stress and ABA treatments and, ectopic expression of it in tobacco leaves caused programmed cell death (34), which is highly similar to leaf senescence, a type of developmental programmed cell death. Later, *Arabidopsis* MAPKKK18 was reported to function in ABA-induced leaf senescence (30). Although ABA-activated MAPKKK18 and transcriptional activation of *MAPKKK18* are ensured by the core PYR/PYL-SnRK2s pathway (35, 36), the direct molecular and genetic evidences linking the transcription of *MAPKKK18* and ABA core signaling is still missing. Thus, we hypothesized that some ABA-related transcription factors (TFs) directly modulate the expression of *MAPKKK18* and regulate leaf senescence. Here, we provide evidences from both molecular biological and genetic analyses to support that three ABF TFs directly regulate the transcription of *MAPKKK18* to mediate leaf senescence and thus we build a link between ABFs and MAPKKK18.

## Results

### The expression of *MAPKKK18* is induced by ABA treatment, osmotic stress, and leaf senescence

**Figure 1:**
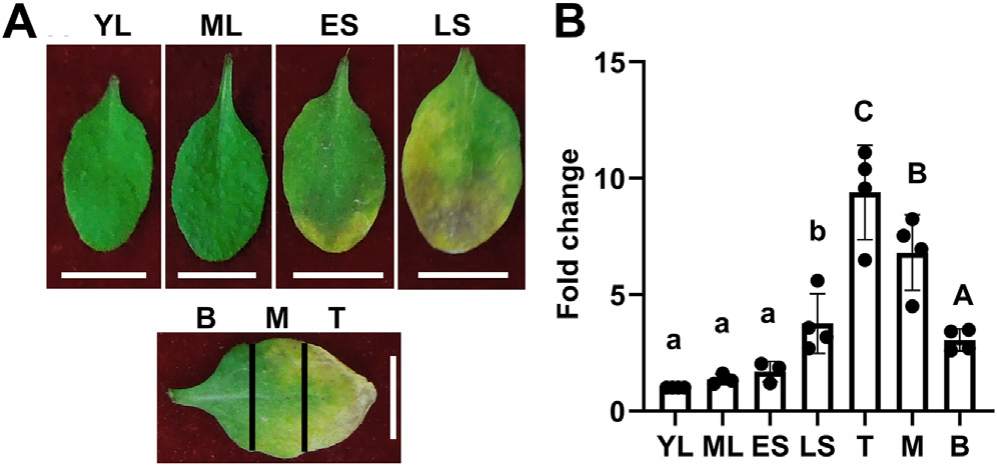
**Leaf senescence induces the expression of *MAPKKK18*.***A*, the development of senescence in rosette leaves at different leaf ages. YL, ML, ES, and LS represents young leaves derived from 3-week-old, mature leaves from 28-day-old, early senescent leaves from 35-day-old plants with less than 25% *yellow area*, and late senescent leaves from 42-day-old with more than 50% *yellow area*, respectively. The tip, middle, and base sections of an early senescing leaf (35 days old) were represented by T, M, and B, respectively. The scale bar represents 1 cm. *B*, the transcription of MAPKKK18 is induced by senescence progress as assayed through quantitative RT-PCR. Data are presented as averages of four biological replicates ±SD. The expression level of MAPKKK18 in YL was arbitrarily set to be 1. *Different letters* indicate significant differences based on one-way ANOVA test followed by Duncan’s multiple comparison test (*p* < 0.05).

We previously employed an array of techniques to elucidate novel functions of MAPKKK members (34, 37). We observed that the *MAPKKK18* expression was induced by ABA treatment and it was also upregulated during the leaf senescence process in *Arabidopsis* (Figs. 1 and S1*A*). Two ABA marker genes, *RD29B (Reponse to Desiccation 29B)* and *RAB18 (Rapid Response to ABA 18)*, were used to monitor ABA responses in wildtype (WT) seedlings and showed expected changes after ABA treatment (Fig. S1*A*). These results indicate MAPKKK18 might play an important role in both ABA responses as well as leaf senescence.

We next examined the subcellular localization of MAPKKK18 *via* a C-terminal GFP fusion. We observed the MAPKKK18–GFP fusion protein emitted green fluorescence signals in nuclei and also in cytoplasm (Fig. S1*B*) and signals in nuclei overlapped well with that of the nuclear marker of NLS-mCherry (Fig. S1*B*). In parallel, a control expressing *GFP* alone exhibited green fluorescence signals in both cytoplasm and nuclei (Fig. S1*B*). Moreover, the MAPKKK18–GFP signals were still observed to be distributed in both the cytoplasm and nuclei in the plasmolyzed tissues after treatment with a hyperosmotic solution (500 mM mannitol) (Fig. S1*B*). We further purified total protein and nuclear protein as well nuclei-depleted fractions expressing *MAPKKK18–GFP* and *GFP* control (Fig. S1*C*). After we performed Western blot analysis using the anti-GFP antibody, we found that both GFP and MAPKKK18–GFP fusion proteins were present in total, cytosolic, and nuclear fractions (Fig. S1*C*), indicating MAPKKK18 is localized in both the cytoplasm and nuclei.

### MAPKKK18 participates in ABA-induced leaf senescence

**Figure 2:**
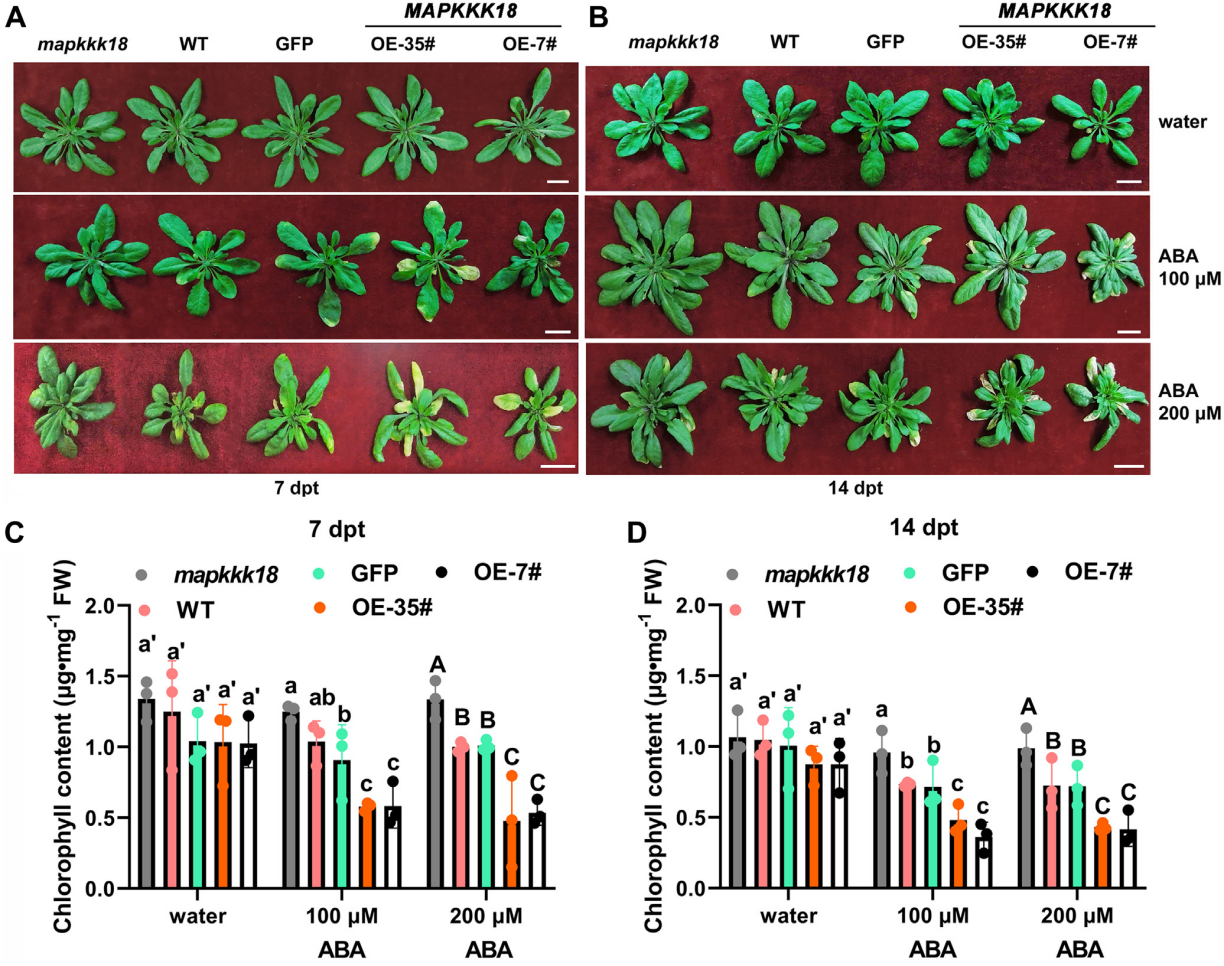
**Comparison of leaf senescence phenotypes of *MAPKKK18* overexpression and mutant lines.***A*, representative plants of 28-day-old soil-grown *mapkkk18*, WT, *MAPKKK18* overexpression (OE-7# and OE-35#), and *GFP* transgenic control after treatment with water or 100 μM or 200 μM ABA for 7 days. The scale bar represents 2 cm. *B*, representative plants of 28-day-old soil-grown *mapkkk18*, WT, *MAPKKK18* overexpression, and *GFP* transgenic control after treatment with water or ABA for 14 days. The scale bar represents 2 cm. *C* and *D*, quantitative comparison of chlorophyll contents among *mapkkk18*, WT, *MAPKKK18* overexpression plants, and *GFP* transgenic control at 7 dpt (*C*) and 14 dpt (*D*). Data shown represent means of three biological replicates ±SD. Different letters indicate significant differences based on one-way ANOVA test followed by Duncan’s multiple comparison test (*p* < 0.05).

Most studies of ABA-induced leaf senescence documented were performed using detached leaves except PYL9 (9). ABA-induced leaf senescence *in planta* would be better as it can reflect the whole status of plants grown in soil. Therefore, we performed an ABA-induced leaf senescence assay *in planta* using T-DNA insertion mutants and constitutive overexpression lines of *MAPKKK18* (Figs. S2 and S3). Four T-DNA insertion mutants were obtained for *MAPKKK18* and homozygous mutants were screened by PCR, with the exact insertion sites of T-DNA among these four mutants identified by sequencing of the flanking sequences. A further semiquantitative RT-PCR analysis showed that GABI_244G02, SALKseq_034842, and SALKseq_123341 are knockout mutants while SALKseq_087047 is a knock-down mutant (Fig. S2, *A* and *B*). Seeds of different mutants and WT were harvested from plants grown under the same normal conditions and at the same period. A phenotypic assay among mutants and WT demonstrated that all four mutants of *MAPKKK18* showed delayed senescence compared with WT (Fig. S2*C*), which was supported by the higher chlorophyll content in mutants than in WT at both 7 and 14 days post treatment (dpt) (Fig. S2*D*). Moreover, two high expression lines, *MAPKKK18-OE-7#* and *OE-35#*, were identified through quantitative RT-PCR (qRT-PCR) (Fig. S3). Similarly, soil-grown *mapkkk18* mutant, WT, *GFP* transgenic control, *MAPKKK18-OE-7#*, and -*OE-35#* were subjected to ABA-induced leaf senescence assay. It can be seen that the *mapkkk18* mutant leaves displayed a stay-green phenotype compared with WT while the two *MAPKKK18* overexpression lines showed accelerated leaf senescence, compared with WT and *GFP* line, at 7 and 14 dpt (Fig. 2, *A* and *B*). Consistent with the visible phenotypic difference, the chlorophyll content of the *mapkkk18* mutant was significantly higher than that of WT and *GFP* line (Fig. 2, *C* and *D*). In contrast, *MAPKKK18*-overexpressing lines exhibited significantly decreased chlorophyll contents compared with WT and *GFP* line (Fig. 2, *C* and *D*). However, under the mock-treated condition, there was no obvious difference among the different genotypes. Therefore, MAPKKK18 positively regulates ABA-induced leaf senescence *in planta*. Our results thus agree well with the detached leaf assay from the other group (30).

Moreover, ABA-induced senescence in detached leaf assay using mutants of *mapkkk18* has not been performed before; we therefore carried out this assay using both overexpression and mutant lines of *MAPKKK18*. The results showed that *mapkkk18* leaves exhibited a stay-green phenotype compared with WT at both 3 and 8 dpt (Fig. S4*A*) and the chlorophyll level in *mapkkk18* mutant leaves was higher than in WT (Fig. S4*B*). In contrast, leaves of overexpression lines of *MAPKKK18* (OE-7# and OE-35#) exhibited a remarkable yellowish phenotype compared with GFP control and WT, when either the sixth and seventh or eighth rosette leaves were used for phenotypic comparison (Fig. S5, *A* and *C*). Consistently, the chlorophyll contents of the OE-7# and OE-35# lines were significantly lower than that of WT or GFP control (Fig. S5, *B* and *D*). These results further support that MAPKKK18 positively regulates ABA-induced leaf senescence (30). We also compared the natural senescence rate between *mapkkk18* mutant and WT plants and found that *mapkkk18* mutant did show delayed leaf senescence compared with WT control at 7-week-old (Fig. S6*A*) and, expectedly, the chlorophyll content of *mapkkk18* was significantly higher than that of WT (Fig. S6*B*). Altogether, our results indicate that MAPKKK18 is required for ABA-induced leaf senescence.

### ABA-induced expression of *MAPKKK18* is abolished in *abf2abf3abf4* triple mutant

**Figure 3:**
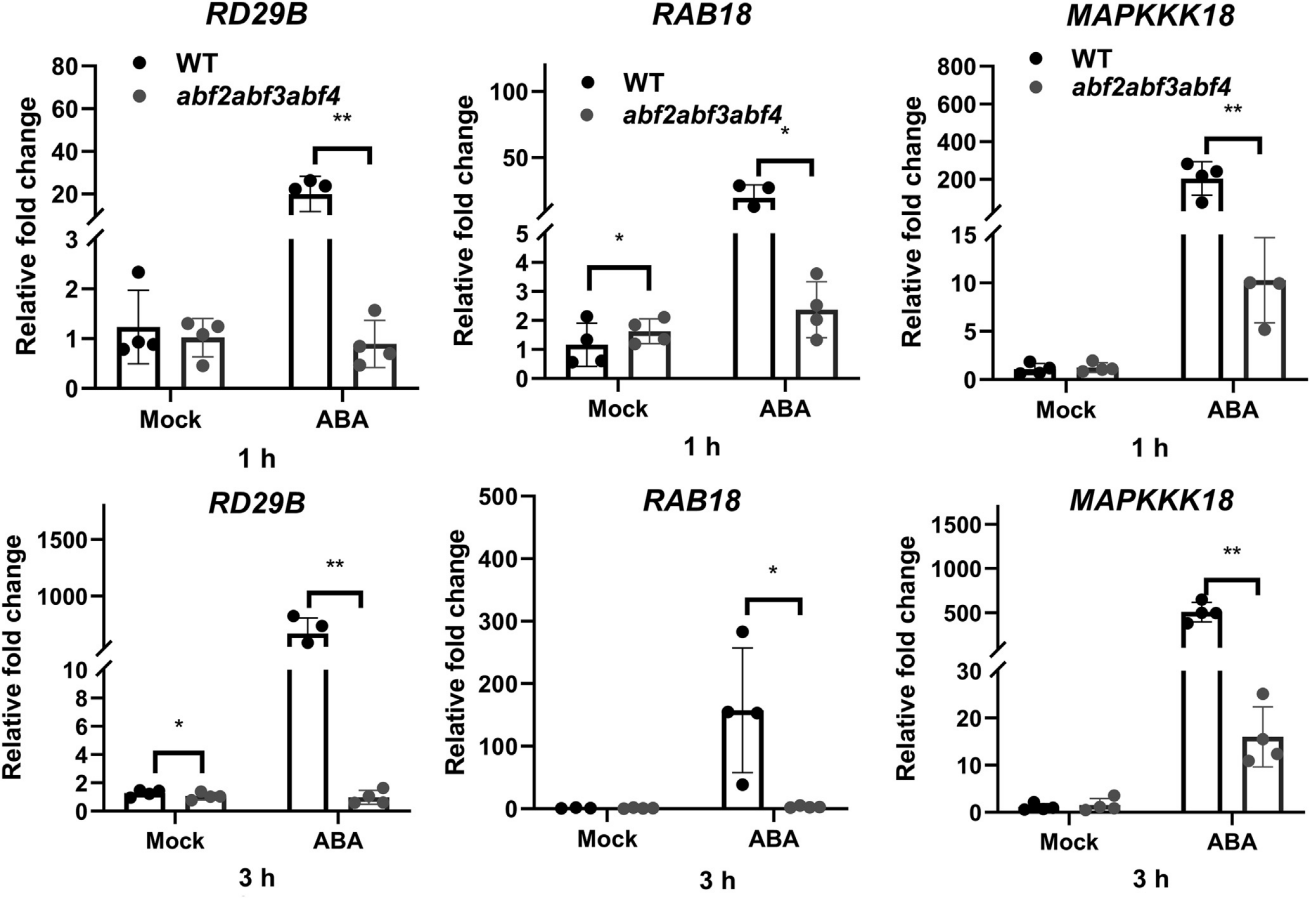
**Analysis of *MAPKKK18* expression in *abf2abf3abf4* triple mutant after ABA treatment.** Analysis of the expression level of *MAPKKK18* in the *abf2abf3abf4* triple mutant after 50 μM ABA treatment for 1 h and 3 h. Expression of *RD29B* and *RAB18*, as ABA-responsive marker genes and direct targets of ABA-responsive element binding factors, was monitored as a control. Data are means of at least three independent biological replicates ±SD. *Asterisks* indicate significant differences between WT and mutants by Student’s *t* test (∗*p* < 0.05; ∗∗*p* < 0.01). ABA, abscisic acid.

We found that *MAPKKK18* was significantly induced by ABA treatment in WT plants during a time-course expression study (Fig. S1*A*). However, its upstream TFs remain to be elucidated. Considering that ABF TFs are key components of ABA signaling and *ABA-Responsive Element* (*ABRE*), which harbors the core sequence of 5′ACGTGGC3′ (or 5′GCCACGT3′ on the antisense strand), was identified in the promoter region of *MAPKKK18* (Fig. S7), we speculated that the transcription of *MAPKKK18* is modulated by ABFs. Thus, the expression level of *MAPKKK18* in *abf* mutants was analyzed. The T-DNA insertion mutants of *abf1*, *abf2*, *abf3*, *abf4*, *areb3*, and *ABA insensitive 5* (*abi5*) were examined by semi-qRT-PCR, and the results showed that *abf1*, *abf4*, and *abi5* were knockout mutants while *abf2*, *abf3*, and *areb3* were knock-down mutants (Fig. S8). A qRT-PCR assay showed that the mRNA level of *MAPKKK18* was significantly decreased in *abf2*, *abf3*, and a*bf4* mutants compared with WT after treatment with ABA for 1 h and expression of *MAPKKK18* was still repressed in the *abf3* mutant after ABA treatment for 3 h (Fig. S9). Furthermore, *MAPKKK18* expression was almost abolished in the triple mutant *abf2abf3abf4* (Fig. 3). Similarly, *RD29B* and *RAB18* were used to monitor ABA responses in WT and mutants and the results showed that, as expected, *RD29B* and *RAB18* were markedly induced in WT upon ABA treatment (Fig. 3). On the contrary, the induction of both marker genes was significantly abolished in *abf2abf3abf4* at both 1 and 3 h proving the efficient interruption of the ABA signaling pathway as a result of simultaneous mutations of *ABF2*, *ABF3*, and *ABF4* (Fig. 3). Besides, the mRNA levels of both marker genes were significantly lower in *abf2* and *abf3* at 1 h while decreased in *abf3* at 3 h (Fig. S9). In contrast, mutations of *ABF1*, *AREB3*, or *ABI5* had no significant effect on the expression of *MAPKKK18* (Fig. S9). Hence, ABF2, -3, and -4 could be the sought upstream TFs responsible for the expression of *MAPKKK18*.

### ABF2, ABF3, and ABF4 bind directly to the promoter of *MAPKKK18*

**Figure 4:**
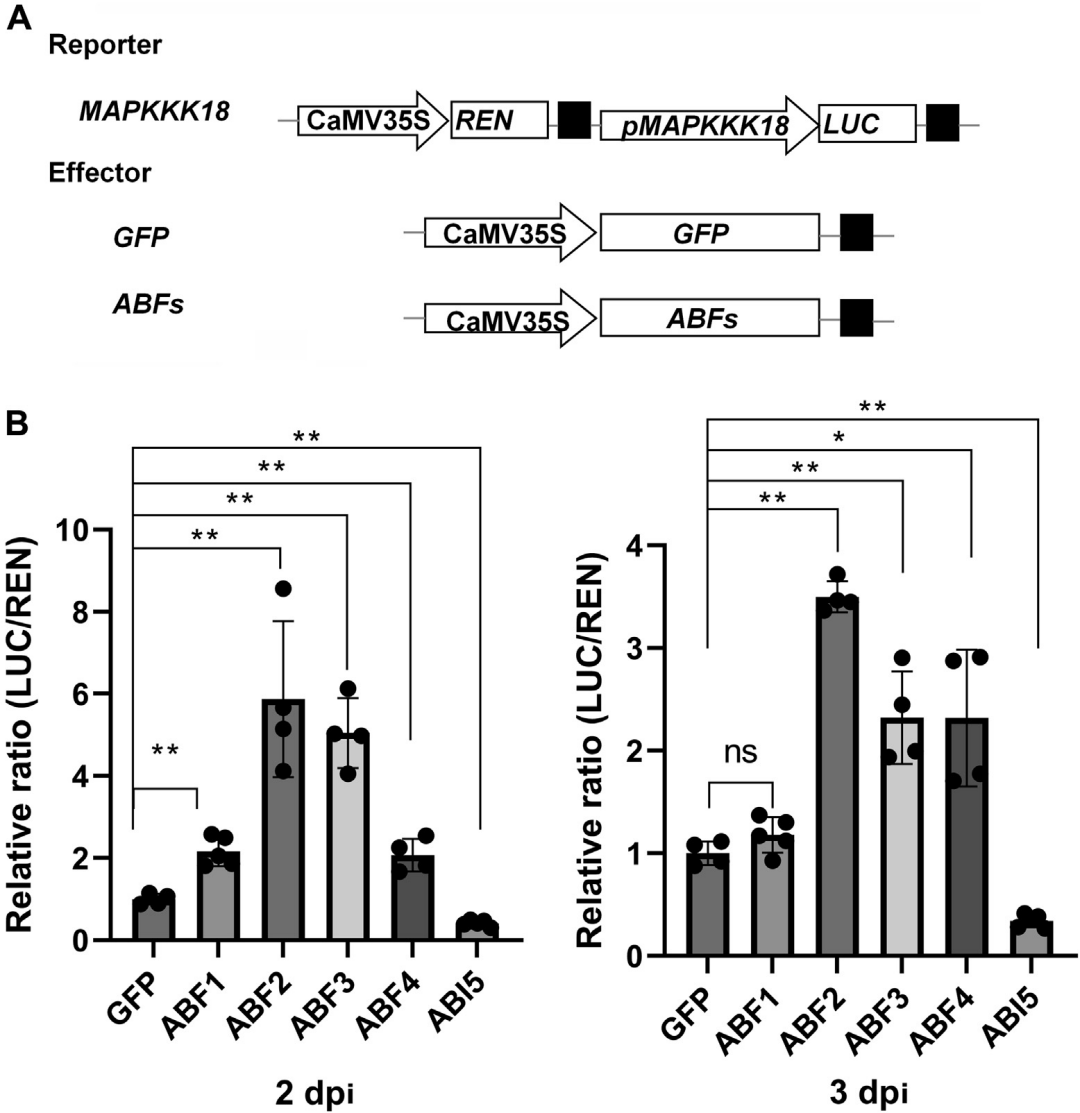
**Multiple ABA-responsive element binding factors (ABFs) transactivate the expression of *MAPKKK18*.***A*, schematic diagrams of reporter and effector plasmids in the dual-luciferase assay. Promoter region of *MAPKKK18* was fused to gene encoding firefly luciferase (LUC). CaMV*35S* promoter-driving gene encoding renilla luciferase (REN) was set as an internal normalization. Effector plasmids are *ABFs/ABI5* or *GFP* genes driven by the CaMV*35S* promoter. *Black box* indicates transcriptional terminator. *B*, the dual LUC assay results. Ratios of LUC/REN were calculated and compared with that of GFP control, which was set as 1. Data are means of four independent biological replicates ±SD. *Asterisks* denote significant differences compared with GFP control by Student’s *t* test (∗*p* < 0.05; ∗∗*p* < 0.01).

We performed a transactivation assay using a dual-luciferase system to further investigate whether the above-identified three ABFs mediate the transcription of *MAPKKK18*. A 2.164-kb region of promoter and 5′ UTR fragment of *MAPKKK18* were cloned upstream of *Firefly Luciferase (LUC)* reporter gene, and *Renilla Luciferase* (*REN*) driven by CaMV*35S* was used as an endogenous control (Fig. 4*A*). The different effectors of *ABFs* driven by CaMV*35S* were individually coinfiltrated with the reporter plasmid into the leaves of tobacco (Fig. 4*A*). A relative LUC activity represented by the LUC/REN ratio was determined and subjected to statistical analysis. It can be seen that significant higher LUC/REN ratios were observed at both 2 and 3 days post infiltration (dpi) when *ABF2*, -*3*, -*4* were individually coexpressed with *ProMAPKKK18::LUC*, whereas *ABF1* showed a significantly higher ratio only at 2 dpi (Fig. 4*B*). On the contrary, ABI5 exhibited repression on the expression of *MAPKKK18* (Fig. 4*B*). Our results thus suggest that only three ABF TFs activate the promoter activity of *MAPKKK18* and hence increase the mRNA level of *LUC* gene driven by the promoter of *MAPKKK18*. We therefore chose ABF2, -3, and -4 for further analysis.

**Figure 5:**
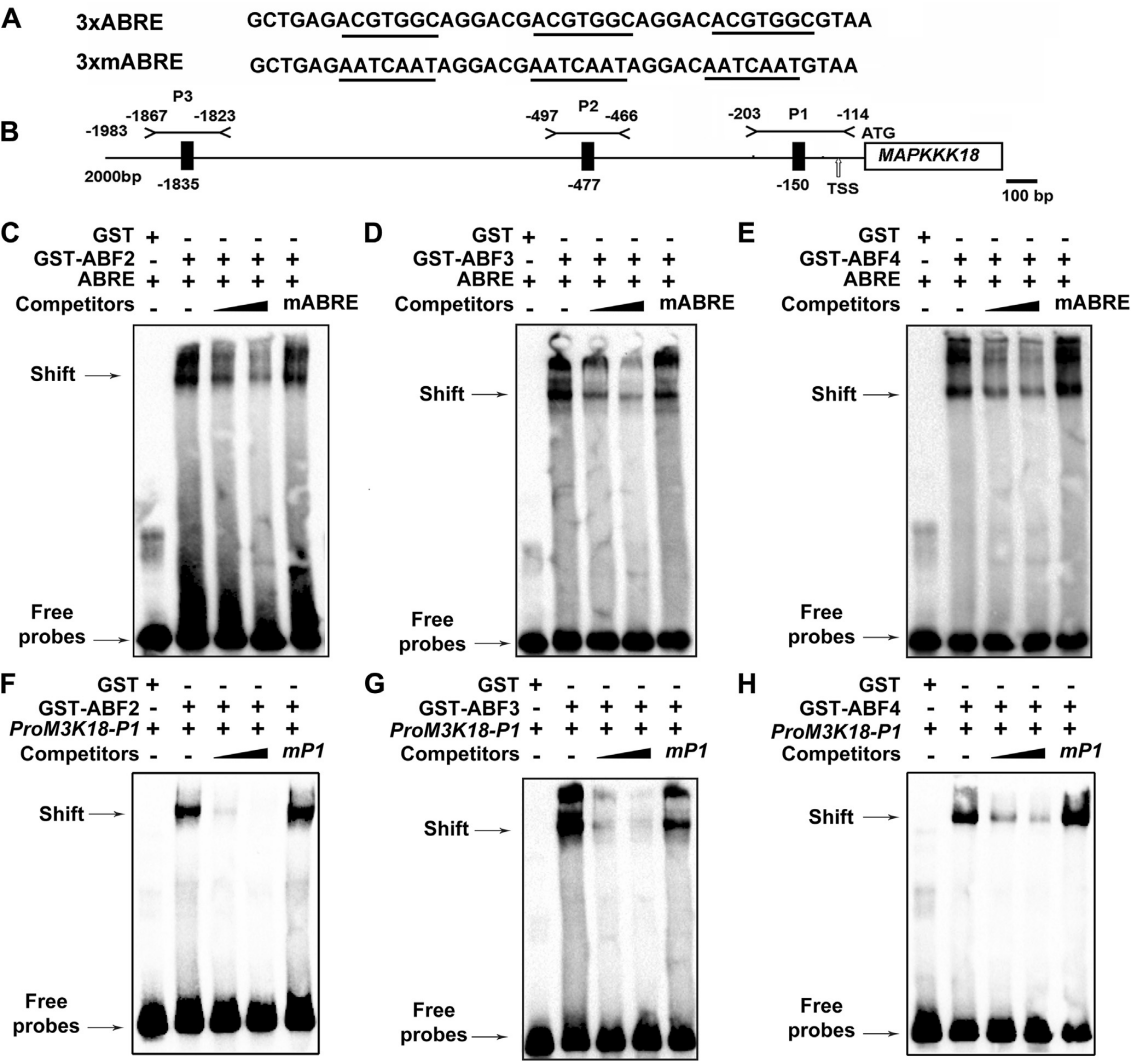
**Abscisic acid–responsive element binding factors physically bind to the promoter region of *MAPKKK18* by EMSA.***A*, the sequences of probes harboring triple tandem repeat of native and mutated ABRE elements used as probes in EMSA. The ABRE and its mutated version were *underlined*. *B*, schematic diagram of distribution of ABRE elements in the promoter region of *MAPKKK18*. *Black rectangles* indicate ABRE elements, and EMSA probes are indicated by *black lines* above the sequence. *Numerals* represent the site upstream of the transcription start site, which is referred to be +1. *C*–*E*, EMSA results of binding of recombinant GST-ABF2 (*C*), GST-ABF3 (*D*), GST-ABF4 (*E*) to native and mutant ABRE elements. *F*–*H*, EMSA results of binding of GST-ABF2 (*F*), GST-ABF3 (*G*), GST-ABF4 (*H*) to the P1 probe of Pro*MAPKKK18* and its mutated form. In *C* through *H*, competitive probes were added in a molar excess as shown above the lanes. mP1 indicates mutated P1 probes. The bands of protein–DNA shift and free probes were indicated by *arrows* at the *upper* and *lower parts* of each image, respectively. “+” and “−” stand for the presence and absence of corresponding components in each binding reaction, respectively. ABRE, abscisic acid–responsive element; EMSA, electrophoresis mobility shift assay.

We performed electrophoretic mobility shift assay (EMSA) assay to examine if ABF2, -3, and -4 could bind to one or more of these ABRE elements in the promoter region of *MAPKKK18*. To this end, fusion proteins of ABF2, -3, and -4 tagged with GST were expressed and purified from *Escherichia coli*. To begin with, we tested the binding specificity of ABF2, -3, and -4 using a triple tandem repeat of ABRE elements (Fig. 5*A*). All of these three ABF TFs can bind to ABRE elements but not to the mutated ABRE elements (Fig. 5, *C*–*E*). Then, to test whether these three ABF TFs bind to the promoter region of *MAPKKK18*, a total of three probes, designated as P1 through P3, were synthesized, which spanned the ABRE elements in the promoter region of *MAPKKK18* (Fig. 5*B*). The binding of ABFs to these three probes was tested one by one. Shifted bands of probes with GST-ABF2, GST-ABF3, or GST-ABF4 were observed (Fig. S10, *A*–*C*), which suggests that ABF2, ABF3, and ABF4 can physically associate with these probes *in vitro*. Moreover, a competitive assay showed that excessive amounts of unlabeled cold probes successfully attenuated the signal of shifts in a dose-dependent manner (Fig. 5, *F*–*H*), which is indicative of binding specificity. Taken together, these data clearly reveal that ABF2, -3, and -4 directly bind to the promoter region of *MAPKKK18*.

**Figure 6:**
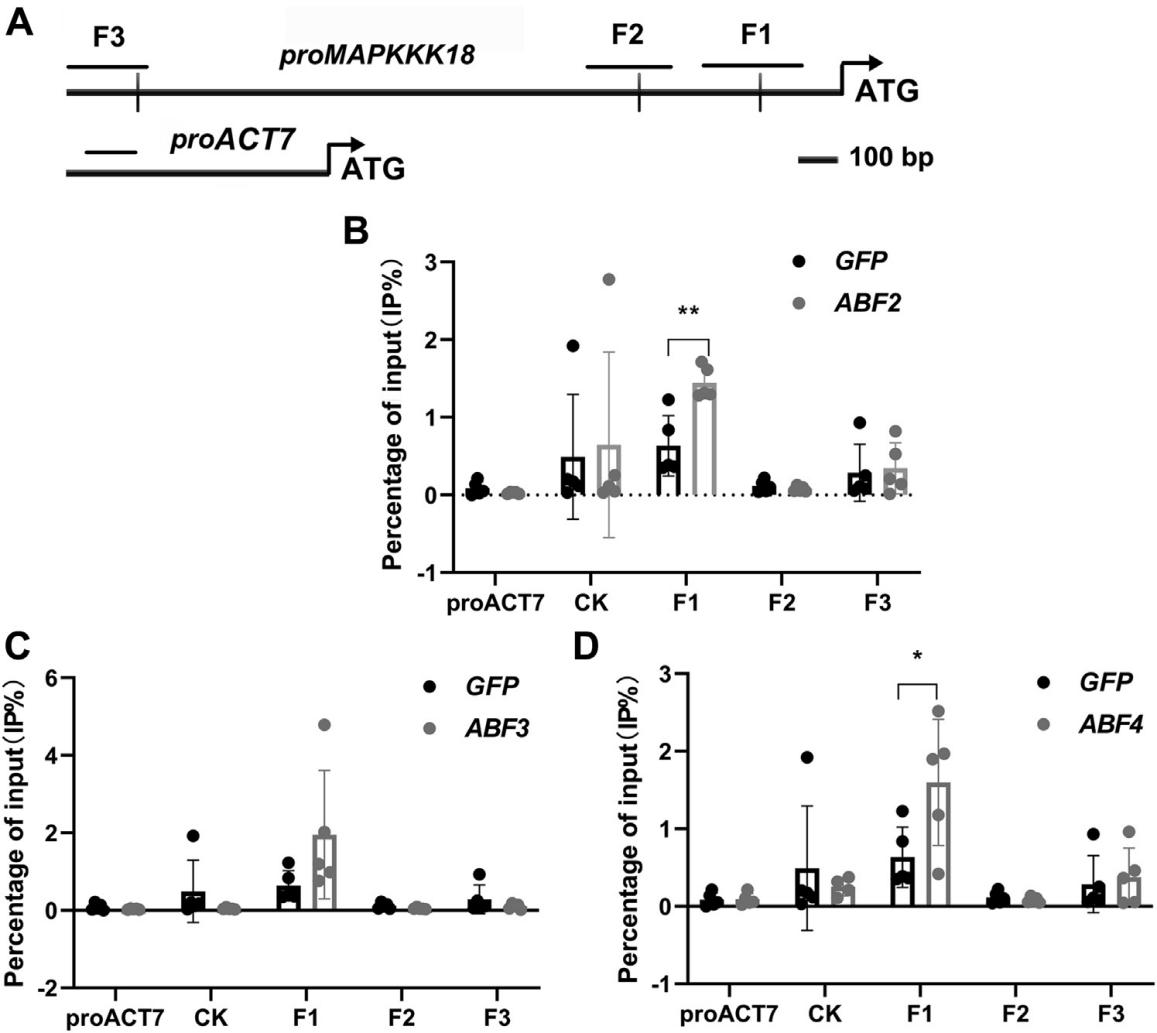
**ABF2, ABF3, and ABF4 directly bind to the promoter of *MAPKKK18* by ChIP assay.***A*, the promoter region of *MAPKKK18* showing the position of abscisic acid–responsive elements (*black vertical line*) and ChIP amplicons (*black horizontal lines*). F1 through F3 represent three regions amplified by quantitative PCR. The *ACT7* promoter region was amplified as a control. *B*–*D*, *in vivo* ChIP assay showing binding of ABF2, -3, and -4 to the promoter of *MAPKKK18* upon abscisic acid treatment. Chromatins were immunoprecipitated from transgenic plants expressing different *ABFs* with *GFP* transgenic line used as a control. Data shown are means of five biological replicates ±SD. A control region (CK) located over 1 kb downstream of the ATG start codon of *MAPKKK18* was amplified as a second control. Significant differences between *ABF* transgenic plants and *GFP* control by Student’s *t* test are indicated with *asterisks* (∗*p* < 0.05; ∗∗*p* < 0.01). ChIP, chromatin immunoprecipitation.

We further employed an *in vivo* chromatin immunoprecipitation (ChIP) quantitative PCR (qPCR) assay to detect the physical association of ABF2, -3, and -4 with the promoter region of *MAPKKK18*. To do so, transgenic plants of *ABF2*, -*3*, and -*4* tagged with an epitope Myc tag were generated and characterized by immunoblotting using the anti-Myc antibody (Fig. S11). Proteins of ABFs and their associated DNA were immunoprecipitated with anti-Myc antibody from transgenic plants (Fig. 6). The enrichment of *MAPKKK18* promoter regions containing ABRE *cis*-elements was quantified through qPCR. Interestingly, we observed a significant 2-fold enrichment of F1 region by ABF2 and -4, but no enrichment of F2 and F3 fragments or the *ACT7* control region (Fig. 6). ABF3 also showed a trend to bind the F1 fragment (Fig. 6). Our results thus demonstrate that ABF2, -3, and -4 bound to ABRE-containing F1 region of the *MAPKKK18 in vivo* (Fig. 6). Furthermore, we examined the expression level of *MAPKKK18* in *ABF4-OE/abf2abf3abf4*, one of the transgenic lines for ChIP-qPCR. We found that the expression level of *MAPKKK18* in *ABF4-OE/abf2abf3abf4* was significantly higher than in *GFP*-OE/*abf2abf3abf4* and *abf2abf3abf4* at both 1 h and 3 h after ABA treatment (Fig. S12). Moreover, when we examined ABA-induced senescence phenotype among WT, *abf2abf3abf4*, and *ABF4*-OE/*abf2abf3abf4*, we observed that the delayed senescence in *abf2abf3abf4* was alleviated in the *ABF4*-OE/*abf2abf3abf4*-23# line used in the ChIP assay (Fig. S13).

### ABA-induced senescence brought about by *ABF2*, *ABF3*, and *ABF4* overexpression is at least partially abolished in the *mapkkk18* mutant background

**Figure 7:**
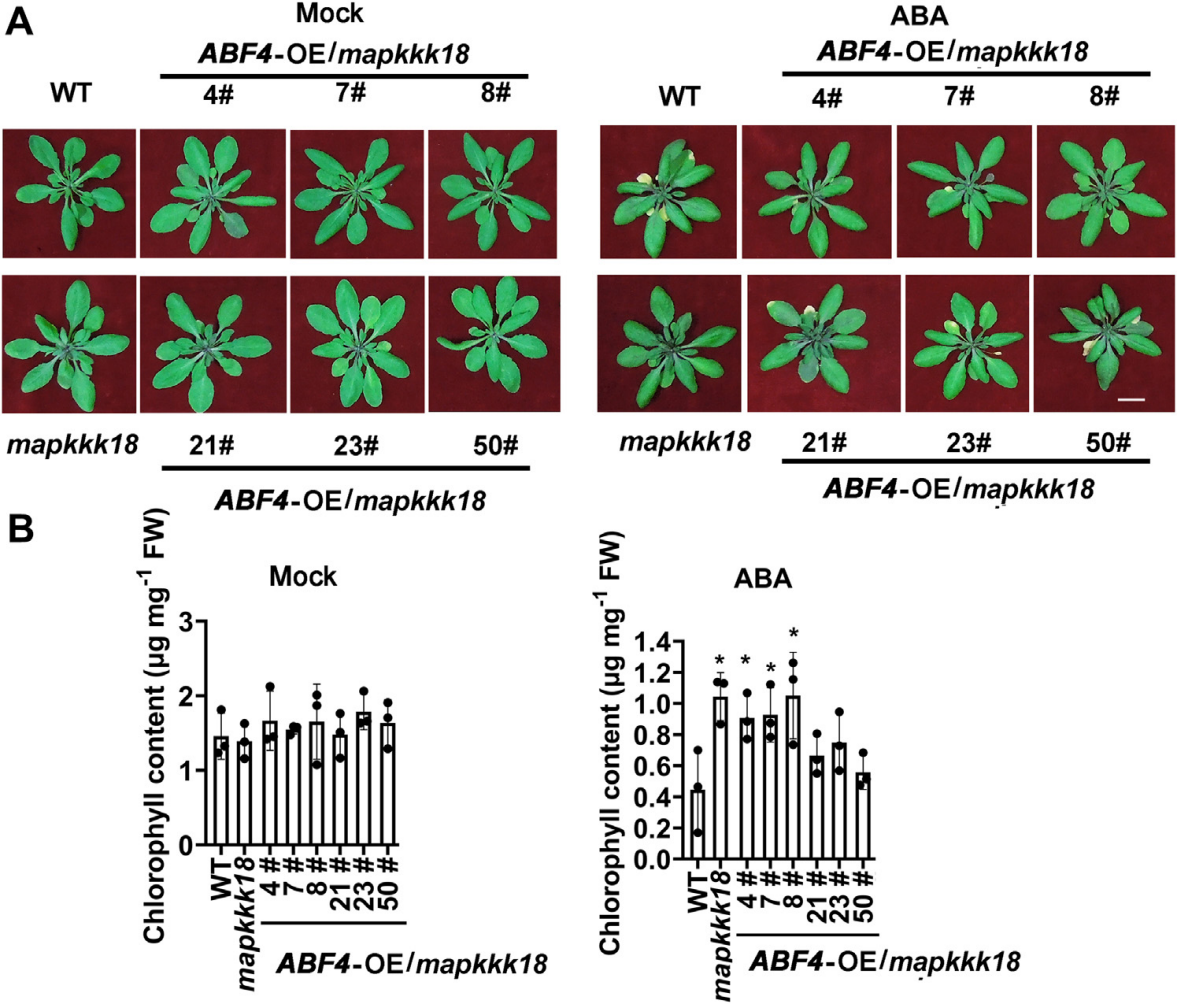
**The precocious senescence of *ABF4* overexpression lines was partially compromised in *mapkkk18*.***A*, six independent transgenic lines of *ABF4-OE mapkkk18* were grown until 26 days old. These images were selected to exhibit more clearly stay-green phenotype out of the full range of 30 independent lines from Fig. S15*A*. *mapkkk18*, *ABF4-OEmapkkk18* lines, and WT were treated without (mock) or with 25 μM abscisic acid (ABA) for 8 days. The scale bar represents 2 cm. *B*, quantitative comparison of chlorophyll contents in rosette leaves of different plants as shown in (*A*). Data are means of three independent biological replicates ±SD. *Asterisks* indicate significant differences compared with WT by Student’s *t* test (∗*p* < 0.05).

As it has been reported that ABF2, ABF3, and ABF4 promote leaf senescence through transactivating chlorophyll catabolic genes *NYE1/SGR1*, *PAO*, and *NYC1* (6) or *SAG29* (9), we further speculated that ABFs-*MAPKKK18* might represent a distinct pathway in leaf senescence. Therefore, we tested whether the absence of *MAPKKK18* will abolish ABA-induced senescence when *ABF2*, *ABF3*, and *ABF4* were overexpressed. We generated overexpression line of *ABFs* in the *mapkkk18* mutant. Transgenic lines showing varied expression levels of *ABFs* were chosen for phenotypic analysis (Fig. S14). A quantification of the chlorophyll content showed that the precocious senescence phenotype induced by *ABF*s overexpression was partially abolished in *ABF2*-OE/*mapkkk18* and *ABF4*-OE/*mapkkk18* lines (Figs. 7, S15 and S16), while it was completely abolished in *ABF3-OE/mapkkk18* lines after treatment with ABA (Fig. S17). Therefore, ABFs promote ABA-induced leaf senescence partially through the downstream target of *MAPKKK18*.

We further generated *ABF4* overexpression transgenic plants in the background of Col-0, and two high expression lines 47# and 48# were selected to examine the phenotype, as a positive control (Fig. S18). It can be seen that, after ABA treatment, *ABF4*-OE/Col-0-47# and 48# lines showed earlier senescence than WT while *ABF4*-OE/*mapkkk18*-4# and 7# lines showed slower senescence than WT (Fig. S19). As a control, the *abf2abf3abf4* triple mutant showed obviously delayed leaf senescence compared with WT (Fig. S19).

We further analyzed the expression of *NYE1*, *NYE2*, *NYC1*, *PAO1*, and *SAG29* in *MAPKKK18*-OE, *mapkkk18*, as well as *ABF4*-OE/*mapkkk18* lines after treatment with ABA. We found that expression levels of *NYE1*, *NYE2*, *NYC1*, and *SAG29* were significantly lower in the *mapkkk18* mutant than in WT at 4 days and all the five genes were repressed in *mapkkk18* at 7 days, after ABA treatment (Fig. S20). On the contrary, *NYE1*, *NYE2*, *NYC1*, *PAO*, and *SAG29* expression levels were significantly higher in at least one of the three *MAPKKK18-OE* lines than in WT (Fig. S20*A*). In addition, *NYE1*, *NYE2*, *NYC1*, *PAO*, and *SAG29* expression was repressed in two *ABF4*-OE/*mapkkk18* lines (#4 and #7) compared with WT at 7 days (Fig. S20*B*), indicating *NYE1*, *NYE2*, *NYC1*, *PAO*, and *SAG29* transcription were compromised by *MAPKKK18* mutation. This result further supports *NYE1/NYE2/NYC1/PAO/SAG29* expression is dependent on the ABFs-*MAPKKK18* pathway.

## Discussion

Leaf senescence is associated with nutrient remobilization from senescing leaves to the storage and developing parts in plants (38). ABA biosynthesis and signaling pathways, triggered or activated mainly by abiotic stresses, play an important role in senescence (11). The significance of the ABA-triggered leaf senescence or abscission helps plants to survive under extreme drought conditions (9).

An ABA signaling module consisting of ABA receptor PYL9, PP2C coreceptors, SnRK2, and ABFs regulates the expression of *SAG29*, etc. to promote leaf senescence (9). SnRK2s phosphorylate ABFs and Related to ABI3/VP1 (RAV1) to positively regulate ABA-induced senescence (6, 39). ABFs also directly regulate chlorophyll catabolic genes (*NYE1*/*SGR1*) and *SAG29* to trigger ABA-induced leaf senescence (6, 9). Besides, several stress-responsive NAC TFs (ANAC019/055/072/002/081/102/032) mediate ABA-triggered leaf senescence, representing a different pathway from that of ABFs (40).

**Figure 8:**
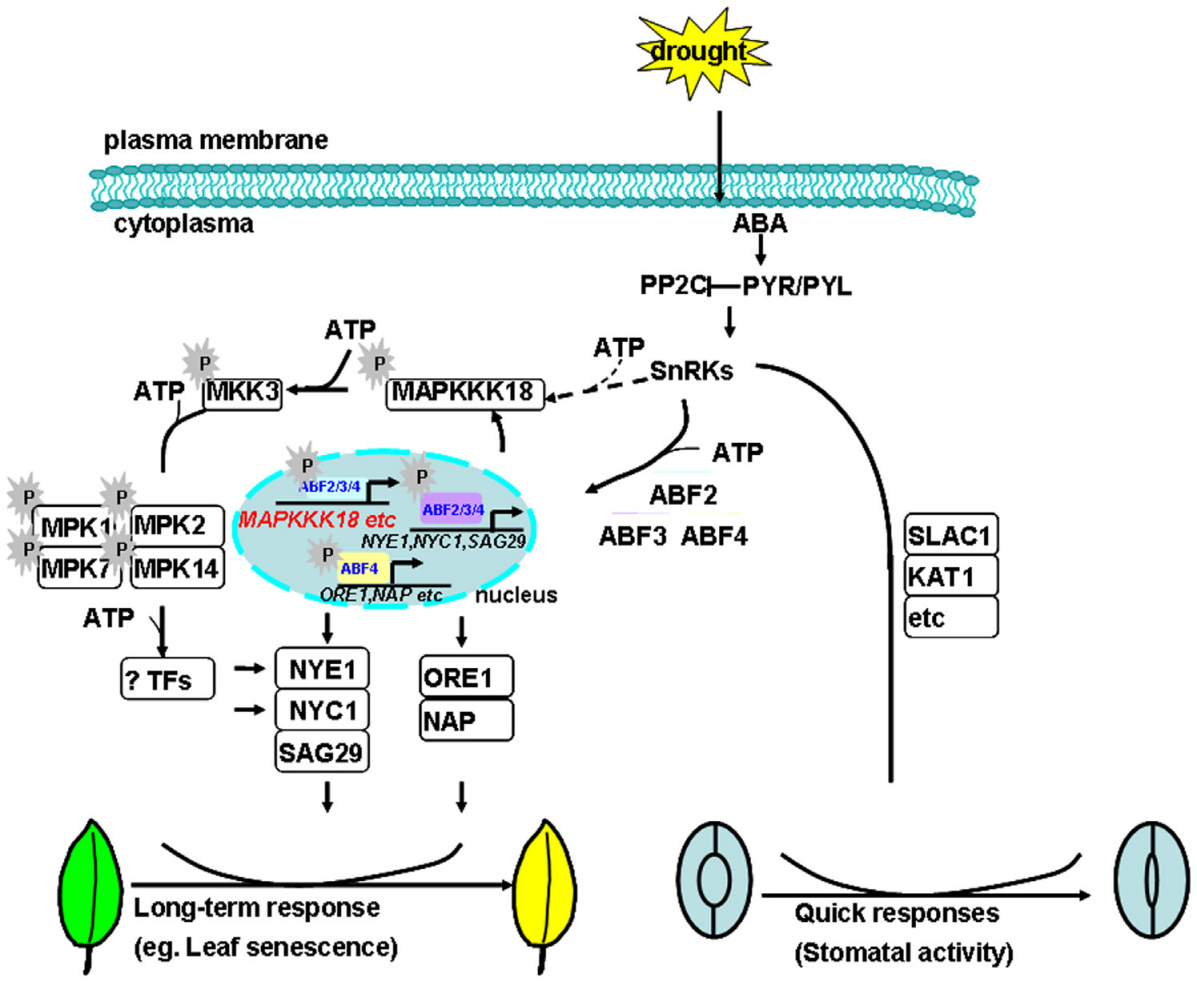
**A working model of transcriptional regulation of *MAPKKK18* by ABFs in ABA-mediated leaf senescence.** ABA accumulates to induce both quick responses such as stomatal closure and long-term responses of plants including leaf senescence and osmotic regulation during drought stress. The core ABA signaling components composed of PYR/PYL-PP2C-SnRK2s were found to activate stomatal closure by phosphorylating SLAC1, KAT1, etc. and therefore reduce the water and turgor loss. ABA induces leaf senescence by ABFs through NYE1 and ORE1 pathways and by a MPK module composed of MAPKKK18-MKK3-MPK1/2/7/14. Here, we proposed an evidence connecting ABF2/3/4 and *MAPKKK18* during ABA-induced leaf senescence. *Solid lines* indicate confirmed regulations as reported in this study and in publications (see the main text), and *dashed lines* with *arrows* imply a possible regulation at the posttranscriptional level. ABA, abscisic acid; ABF, abscisic acid–responsive element binding factor.

So far, only a few members of the MAPK cascade have been identified to be involved in ABA response including seed germination and guard cell signaling (41, 42). They are MPK9 and MPK12 (43), MKK1-MPK6 (44, 45), MAPKKK18 (30, 31, 35) and MAPKKK20 (33). Of them, MAPKKK18, together with MAPKKK17, recruits MKK3-MPK1/2/7/14 to mediate ABA-mediated response, and MAPKKK18 was identified to positively regulate ABA-mediated leaf senescence (30, 35). Its *de novo* protein synthesis, phosphorylation, and activation are controlled by the PYR/PYL-SnRK2 module after ABA treatment (35) whereas MAPKKK18 is dephosphorylated and inactivated by ABI1 for turnover *via* the proteasome pathway in the absence of ABA (31, 36) (Fig. 8). Transcriptional upregulation of *MAPKKKs* by ABA signaling components is possibly required to form a forward feedback loop to ensure plants that grow under prevailing stress conditions acquire a persistent adaptation (35). However, the direct upstream factor(s) initiating the transcription of *MAPKKK18* has been unknown. Here, we present a direct link between ABFs and *MAPKKK18* for ABA-induced leaf senescence.

We found that MAPKKK18 is a regular kinase without any transmembrane domain and was localized in both cytoplasm and nuclei (Fig. S1*B*), which was confirmed by an immunoblotting analysis (Fig. S1*C*). This result is slightly different from a previous report, which found that MKKK18-GFP was predominantly localized in the nucleus of *Arabidopsis* protoplasts (31). We further identified a few *ABRE* elements in the promoter region of *MAPKKK18* (Fig. S7), which may explain why *MAPKKK18* is induced by ABA (Fig. S1*A*), since a previous study indicates that a single copy of ABRE is not sufficient for ABA-responsive transcription (19). Expression of *MAPKKK18* was significantly repressed in *abf2*, *abf3*, and *abf2abf3abf4* triple mutants (Fig. 3). Further analysis through EMSA and ChIP-qPCR as well as transactivation assays revealed that ABF2, -3, and -4 can directly bind to the promoter of *MAPKKK18* and enhance its transcription (Figure 4, Figure 5, Figure 6). These results demonstrate the core TFs of ABA signaling directly regulate *MAPKKK18* expression. Moreover, since ABFs and MAPKKK18 exert a similar function in ABA-induced leaf senescence (6, 30), this study has thus established a link between them. A further genetic study revealed that, in the absence of *MAPKKK18*, ABA-induced senescence rate in *ABFs*-OE/*mapkkk18* plants was intermediate to WT and *mapkkk18* (Figs. 7 and S15–S17). This observation that the attenuation of leaf senescence phenotypes as a result of *ABF2/3/4* overexpression in *mapkkk18* background suggests that ABF2/3/4 act through and independent of *MAPKKK18* in regulating leaf senescence.

Compared with the already known targets activated by ABF2, -3, and -4 in ABA-induced leaf senescence (6, 9), we provide another possible parallel pathway, which is right through promoting the expression level of *MAPKKK18* (Fig. 8).

It is known that ABA accumulates to induce both quick responses such as stomatal closure and long-term responses including leaf senescence and osmotic regulation during drought stress (46) (Fig. 8). Stomatal closure is activated by phosphorylating Slow Anion Channel-associated 1 (SLAC1), K^+^ channel in Arabidopsis 1 (KAT1), etc., through the core ABA signaling components composed of PYR/PYLs-PP2Cs-SnRK2s and therefore reduce the water and turgor loss (46) (Fig. 8). As for the mechanism of the long-lasting response induced by ABA, for example, ABA-induced leaf senescence is mediated through ABFs-*NYE1* (6), -*ORE1* pathways (9), and is through integrating a complete MPK module composed of MAPKKK18-MKK3-MPK1/2/7/14 (35) (Fig. 8). However, most studies focus on the phosphorylation and activation of MAPK members. The importance of the transcriptional regulation of MAPK members during plant growth is seldom discussed.

It is reported that ABA activates phosphorylation of MAPKKK18, possibly by SnRK2.6, with a peak at 30 min, a relatively faster response (30). However, ABA-induced transcriptional activation of *MAPKKK18* occurs at 2 h after ABA treatment in 10-day-old seedlings (35) and 30 min after ABA treatment in 14-day-old seedlings (31). Therefore, the MAPKKK18 in the ABA signaling pathway might be regulated at a dual level. This dual-level regulation of a protein, direct regulation of protein stability/activity, and indirect regulation of expression level by the same MPK has been reported before (29). A well-known example is the regulation of the rate-limiting enzymes of 1-aminocyclopropane-1-carboxylic acid synthase (ACS) 2/6 involved in ethylene biosynthesis by MPK3/6 in *Arabidopsis*. MPK3/MPK6 can phosphorylate ACS2/6 to increase their protein stability and therefore increase ethylene production (47, 48). Meanwhile, MPK3/6 phosphorylate WRKY33 TF to activate the expression of *ACS2*/*ACS6*, contributing to a higher ethylene production (49). Thus the dual-level regulation of ACS2/6 by the MPK3/MPK6-WRKY33 module at both transcriptional and posttranslational levels plays a crucial role in determining the kinetics and magnitude of ethylene production. Similarly, this dual level of regulation of MAPKKK18, two independent and interdependent time-dependent signaling cascades, might play an important role for determining the kinetics and magnitude of the ABA signaling pathway (Fig. 8).

In summary, we have provided new evidences in connecting ABF2/3/4 and *MAPKKK18* during ABA-induced leaf senescence. In this model, the gradually increased *MAPKKK18* expression might provide more mRNA for protein translation and, therefore, make the long-lasting response possible. Our model demonstrates the importance of the transcriptional regulation of *MAPKKK18* to the prolonged response governed by ABA (Fig. 8). Moreover, downstream targets of the MAPKKK18–MKK3–MPK7 module are still waiting to be uncovered.

## Experimental procedures

### Plant materials and growth conditions

*Arabidopsis* ecotype Col-0 was used as the wildtype (WT). The T-DNA insertion lines of *mapkkk18* (GABI_244G02, SALKseq_034842, SALKseq_087047, and SALKseq_123341), *abf1* (SALK_132819), *abf3* (SALK_096965), *abf4* (Salk_069523), *areb3* (SALK_061607), and *abi5* (SALK_013163) were obtained from Arabidopsis Biological Resource Center (ABRC) and Nottingham Arabidopsis Source Center (NASC). The *abf2abf3abf4* triple mutant was supplied by Prof. Yamaguchi-Shinozaki (50). *abf2* (SALK_002984) was provided by Prof. Jigang Li. All *Arabidopsis* seeds were surface sterilized with diluted bleach before being sown on half strength Murashige and Skoog (1/2 MS, Caisson Labs) medium (2.15 g l^−1^ basal salts, 1% sucrose, pH 5.7, and 8 g l^−1^ phytoblend). After stratification at 4 °C for 2 days, seed plates were transferred to the growth chamber for germination. Seven-day-old seedlings were transferred into a commercial soil mix (Pindstrup). The growth condition was a photoperiod of 14-h light/10-h dark with a light intensity of 100 ∼ 150 μmol m^−^ s^−1^ unless specified otherwise. Temperature was 20 to 22 °C during daytime/18 to 20 °C at night.

### ABA-induced senescence assay

The same batch of seeds from various genotypes were harvested from plants grown under the same conditions and at the same time for phenotypic assay. ABA-induced leaf senescence *in planta* was performed as described (9). The rosette leaves from 4-week-old soil-grown *Arabidopsis* plants were sprayed with 100, 200 μM ABA (Sigma-Aldrich) once a day for 6 and 8 days. Leaves treated with distilled water were used as the control. Plants were then kept under normal growth conditions and photographed. Contents of chlorophyll in the seventh and eighth true leaves were determined as described (51, 52). Briefly, leaves were incubated with absolute ethanol overnight before the absorbance was measured at 649 and 665 nm in a spectrophotometer (Aoyi). The content of chlorophyll was calculated using a formula of (6.63*A*_665_ + 18.08*A*_649_) × volume mg^−1^ fresh weight.

### qRT-PCR assay

The sixth true leaves derived from 21-, 28-, 35-, and 42-day-old soil-grown *Arabidopsis* Col-0 plants were collected for RNA extraction. Also, the tip, middle, and base sections of an early senescing leaf derived from the sixth true leaves of 35-day-old plants were harvested for RNA extraction using Plant RNA kit (Omega Bio-tek) with on-column DNA digestion.

The first-strand cDNAs were synthesized from 2.5 μg of RNA using an oligo(dT)_18_ and RNase H-MMLV (TaKaRa). After that, qRT-PCR was performed as described (53) using 10-fold diluted cDNAs and a SYBR Green I kit (CWBIO) on a CFX96 real-time PCR machine (Bio-Rad). *Ubiquitin Conjugating Enzyme 21* (*UBC21*) and *Polyubiquitin 10* (*UBQ10*) were used as reference genes for normalization (53). The geometric mean of fold change was calculated as described (53, 54). Primers were listed in Table S1.

Twelve-day-old seedlings vertically grown on 1/2× MS medium were transferred into water overnight for adaptation before treatment with 50 μM ABA in distilled water. Samples were harvested at 1 h and 3 h after ABA treatment. Total RNA was extracted from the seedlings *via* Plant RNA kit (Omega Bio-tek) for examining the transcript levels of *MAPKKK18*, *RD29B*, and *RAB18*.

### Dual-luciferase reporter assay

A 2.164-kb DNA fragment upstream of the initiation codon of *MAPKKK18* including the 5′ untranslated region (5′ UTR) was amplified from *Arabidopsis* genomic DNA using primers listed in Table S1. *ProMAPKKK18* was cloned upstream of the gene encoding firefly luciferase (LUC) in the pGreenII0800-LUC vector. As an endogenous control, *REN* encoding Renilla luciferase driven by the CaMV*35S* promoter was used. The effector plasmids were p35SFC-*ABF1*, -*ABF2*, -*ABF3*, -*ABF4*, and -*ABI5*, and the control effector plasmid was pYJHA-*GFP*. Both effector and reporter plasmids were transformed into GV3101, and different combinations of the effector and reporter plasmids were infiltrated into leaves of 30-day-old *Nicotiana benthamiana*. ABA, 10 μM, was infiltrated into the same site 5 h before leaf discs were harvested at 2 dpi and 3 dpi. A dual-LUC assay kit (Promega) was used to determine the relative enzymatic activity as described (55). The ratio of LUC to REN indicated the transcriptional activities of *ProMAPKKK18* by various effectors. Four independent biological replicates were conducted.

### Electrophoretic mobility shift assay

EMSA was performed as described with a LightShift Chemiluminescence kit (Thermo Scientific) (53). Briefly, primers of three probes harboring ABRE elements in the promoter region of *MAPKKK18* were synthesized and labeled with biotin at the 5′ end (Sangon; Table S1 and Fig. S7). In the binding reactions, 0.5 to 2 μg purified GST-ABFs were incubated with 50 fmol biotin-labeled probes and 200 ng poly (dI•dC) for 20 min at room temperature. Reactions were stopped by adding a loading buffer. Samples were resolved on a 6% native polyacrylamide gel electrophorized in 0.5× Tris-borate-EDTA (TBE) buffer. The biotin-labeled DNA–protein complex was then transferred onto Hybond N^+^ nylon membrane (Amersham) for detection on a ChemiDoc XRS+ system (Bio-Rad).

### Chromatin immunoprecipitation coupled with qPCR

The ChIP assay was performed as described (56). The 5′ end of *ABFs* was fused with 3xMyc tag under the control of the CaMV*35S* promoter in the pFXMyc vector. Myc-GFP was used as a negative control. Basically, 10-day-old seedlings of ABF transgenic plants were treated with 50 μM ABA plus 10 μM MG132 (Sigma) solution for 12 h at dim light of 30 ∼ 40 μmol m^−2^ s^−1^. Nontreated plants were used as the negative control. *ACT7* was used as a nonbinding control (57). Three to five grams of seedlings were fixed with 1% formaldehyde followed with quenching by 0.125 M glycine. Chromatin was then isolated for sonication to achieve 300- to 500-bp fragments. Protein A-agarose magnetic beads (Upstate) were added to preclear the chromatin supernatant. The fragmented chromatin was then immunoprecipitated with protein A agarose beads conjugated with anti-Myc monoclonal antibodies (Cata #MA1980, Invitrogen). Immunoprecipitated chromatin was washed with TE buffer containing 10 mM Tris-HCl (pH8.0), 1 mM ethylenediaminetetraacetic acid (EDTA) followed by two rounds of washing using low salt (150 mM NaCl, 2 mM EDTA, 20 mM Tris-HCl, pH8.0, 0.2% sodium dodecyl sulfate (SDS), 0.5% Triton X-100), high salt (500 mM NaCl, 2 mM EDTA, 20 mM Tris-HCl, pH8.0; 0.2% SDS, 0.5% Triton X-100), and LiCl washing buffer (0.25 M LiCl, 1 mM EDTA, 1% SDS, 10 mM Tris-HCl, pH8.0, 1% NP-40). Chromatin was then eluted with an elution buffer containing 0.1 M NaHCO_3_ and 1% SDS. Afterward, the eluted chromatin was reverse cross-linked at 65 °C overnight and the protein and RNA in the complex were digested with Proteinase K (10 mg/ml; Sigma) and RNaseA, respectively at 45 °C for 2.5 h. The DNA was subsequently isolated by phenol/chloroform extractions for further precipitation by ethanol and glycogen (Fermentas). qPCR was then performed with DNA obtained and primer sets corresponding to overlapping *ABRE* regions of the *MAPKKK18* promoter (Fig. S7). qPCR with primers specific for the promoter of the *ACT7* gene and 1 kb downstream of the start codon of *MAPKKK18* were used as negative controls (Fig. S7 and Table S1). The input is from another aliquot of the supernatant with the same procedure operations, which stands for the equal amount of chromatin used for immunoprecipitation. The fold enrichment is calculated by formula of percentage of input (% of input), which is equal to 100 × 2^Δ(ct(input) − ct(sample))^ × 0.05. The value of 0.05 is the percentage of volume of 50 μl of sonicated chromatin as an input out of a total of 1000 μl. Mock samples (mock-*ACT7*, mock-F1, mock-F2, mock-F3) were individually set to 1, and ABA-treated samples were normalized by individual mocks. Grubb’s test was used to determine the outliers followed with removal from the dataset. Five biological replicates were analyzed. Significant differences were denoted by asterisks through Student’s *t* test compared with the GFP control (∗*p* < 0.05; ∗∗ *p* < 0.01).

### Statistical analysis

All data are presented as means of at least three independent biological replicates ±SD. The data were analyzed with one-way ANOVA followed by Ducan’s multiple comparison tests or Student’s *t* test. Graphs were edited in Photoshop CS (Adobe) or plotted in Sigmaplot 12.3 (Systat Software).

## Data availability

All data are included in the article.

## Supporting information

This article contains supporting information (6, 53, 55, 58, 59, 60).

## Conflict of interest

The authors declare that they have no conflicts of interest with the contents of this article.
