# Supplementary material for: The abscisic acid–responsive element binding factors MAPKKK18 module regulates abscisic acid–induced leaf senescence in Arabidopsis
Source: J Biol Chem. 2023 Feb 24;299(4):103060. doi: 10.1016/j.jbc.2023.103060 (PMC10166789; doi:10.1016/j.jbc.2023.103060)
Supplement: Supplemental Figures S1–S20 and Table S1 [file mmc2.pdf]

The Abscisic Acid-Responsive Element Binding Factors (ABFs)-*MAPKKK18* module mediates ABA- induced leaf senescence in Arabidopsis

Guoying Zhao[1+], Qian Cheng[1+], Yuting Zhao[1], Feifei Wu[1], Bangbang Mu[1], Jiping Gao[2] , Liu Yang[1], Jingli Yan[1], Hanfeng Zhang[1], Xing Cui[1], Qinqin Chen[1], Fangxiao Lu[1], Qianqian Ao[1], Asma Amdouni[1], Yuan-Qing Jiang[1*], Bo Yang[1*]

[1] State Key Laboratory of Crop Stress Biology for Arid Areas, College of Life Sciences, Northwest A & F University, Yangling, Shaanxi, 712100, China
[2] National Key Laboratory of Plant Molecular Genetics, Shanghai Institute of Plant Physiology and Ecology, Chinese Academic of Sciences, Shanghai 200032, China

Running title: ABFs regulate *MAPKKK18* expression in leaf senescence

[+] These authors contributed equally to this work.

[*]To whom correspondence should be addressed: Bo Yang-yangwl@nwafu.edu.cn; Yuan-Qing Jiang - jiangyq@nwafu.edu.cn; College of Life Sciences, Northwest A & F University, Yangling, Shaanxi, 712100, Fax: 86-29-87092262

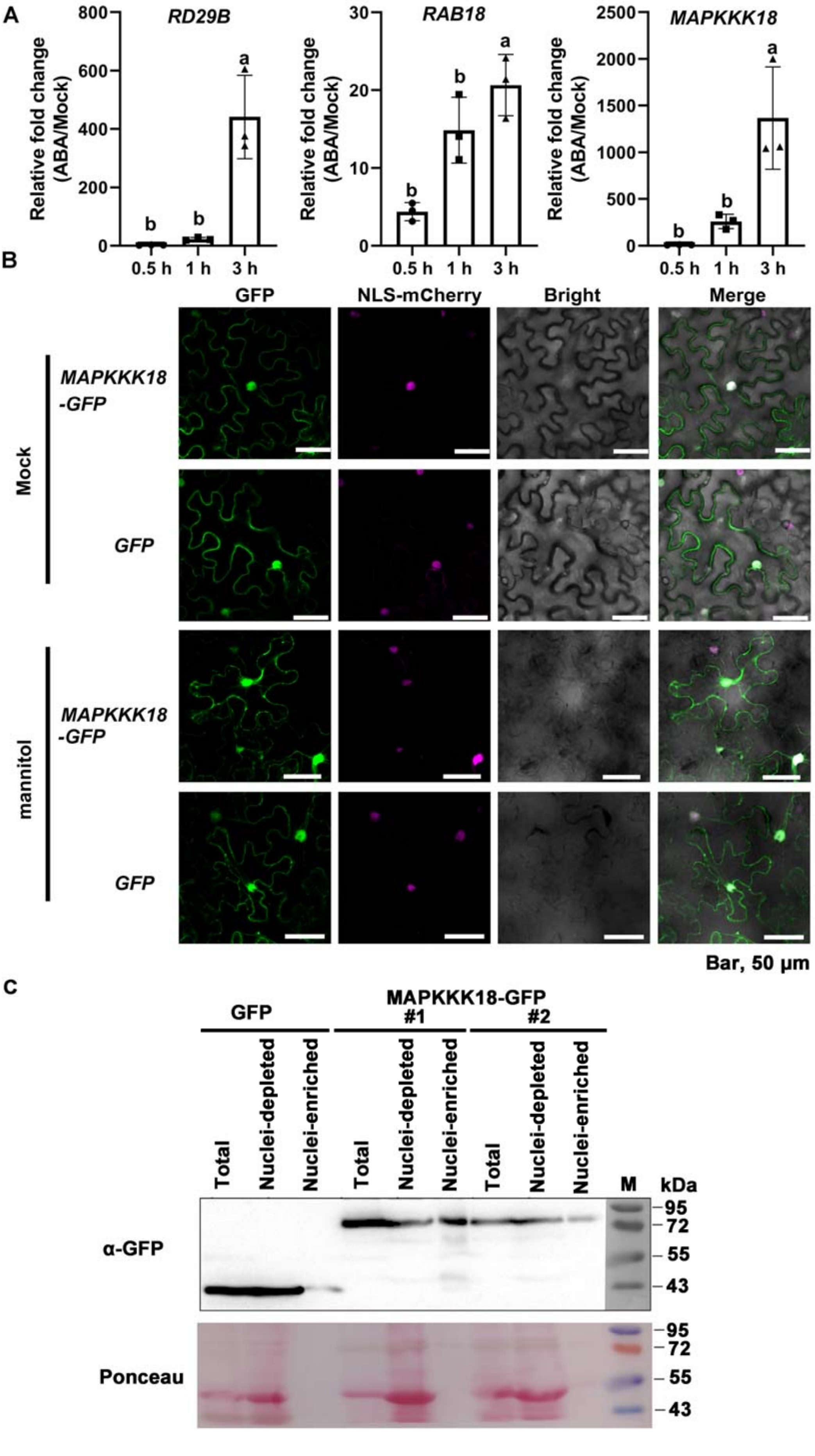

A
RD29B
RAB18
MAPKKK18
Relative fold change (ABA/Mock)
0.5 h
1 h
3 h
B
GFP
NLS-mCherry
Bright
Merge
Mock
mannitol
MAPKKK18 -GFP
GFP
Bar, 50 μm
C
GFP
MAPKKK18-GFP
#1
#2
Total
Nuclei-depleted
Nuclei-enriched
M
kDa
α-GFP
Ponceau
95
72
55
43

Figure S1. Expression analysis and subcellular localization of MAPKKK18. (A) ABA induced the expression of *MAPKKK18* and two marker genes *RD29B* and *RAB18*. 12-d-old seedlings were treated with 50 μM ABA for 0.5, 1 and 3 h. Seedlings grown on 1/2 MS agar were served as a control. The transcript level of *MAPKKK18* was examined through quantitative RT-PCR (qRT-PCR). Data are presented as averages of three biological replicates ±SD. The expression level for each gene was compared with corresponding mock controls at different time points, and different letters indicate significant difference based on one-way ANOVA test followed by Duncan's multiple comparison test ($P<0.05$). (B) Subcellular localization of MAPKKK18 in *Nicotiana benthamiana* epidermal cells. The leaves were agroinfiltrated to express *MAPKKK18-GFP* or *GFP*. The nuclear marker of NLS-mCherry was co-infiltrated in epidermal cells. Leaf discs were further treated with a hyperosmotic solution (500 mM mannitol) for 1 h to induce plasmolysis. The florescence signals were observed under same settings on a confocal microscope. Scale bars, 50 μm. (C) Western blotting analysis of MAPKKK18-GFP and GFP in total protein, nuclei-depleted protein and nuclei-enriched protein from (B) using anti-GFP antibody. The R1 and R2 indicate two independent biological replicates with similar results. The molecular weight of each band of protein marker is indicated at the right side. T, C, N represents total protein, nuclei-depleted protein, and nuclei-enriched protein, respectively. Ponceau S staining of the large subunit of Rubisco was performed as the loading control.

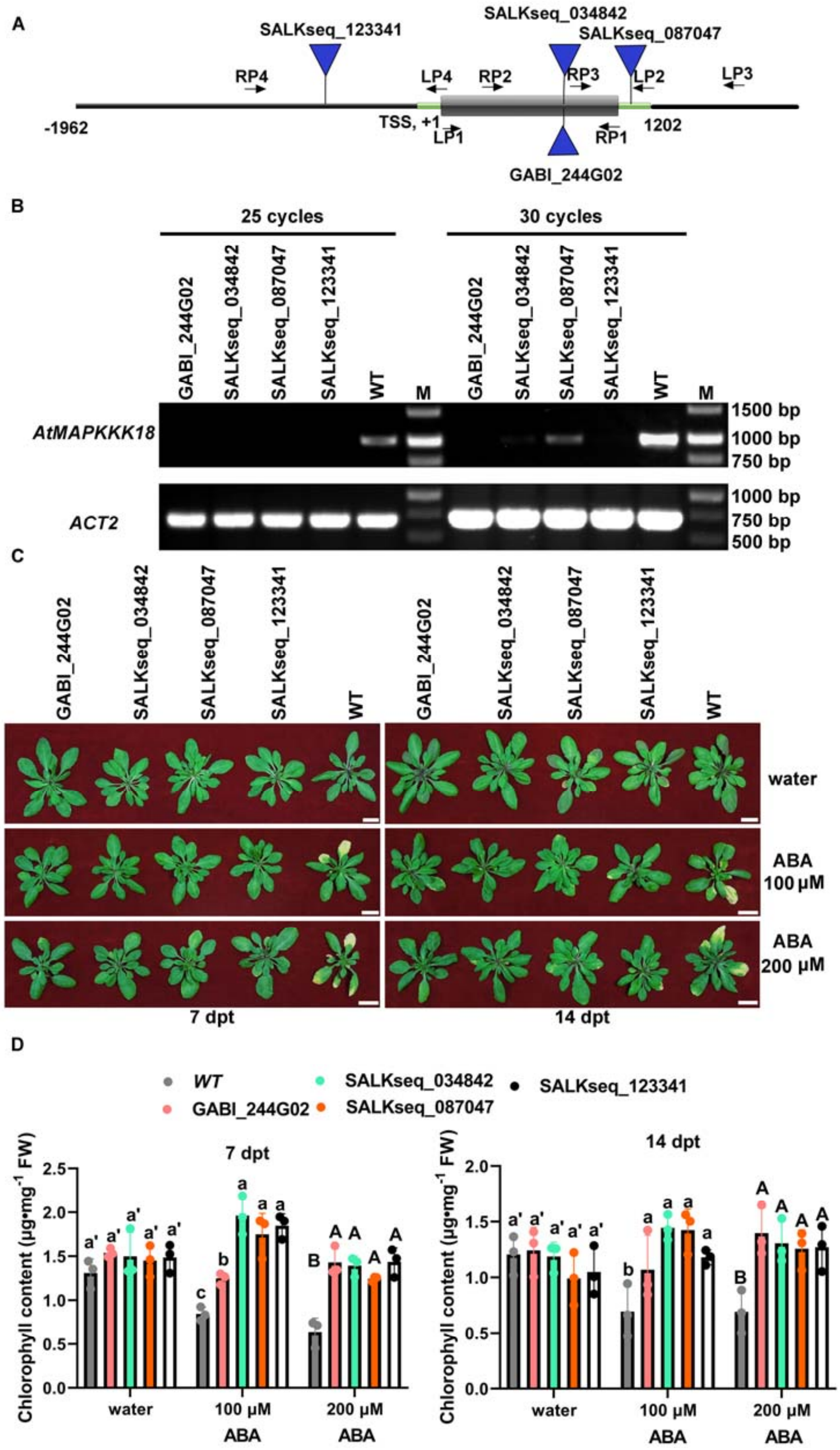

Figure S2. Identification of *mapkkk18* mutants and phenotypic analysis in ABA-induced leaf senescence. (A) The insertion sites of T-DNA region in the genomic DNA of *MAPKKK18*. Four mutants were used and they are GABI_244G02,

SALKseq_034842, SALKseq_087047 and SALKseq_123341. The dark grey rectangle indicates the exon, triangles represent the T-DNA insertion sites, black lines represent promoter or intergenic region while green lines are untranslated regions (UTRs). Arrows represent locations of different primers used for PCR-based genotyping. LP1 and RP1 are for GABI_244G02, LP2 and RP2 for SALKseq_034842, LP3 and RP3 for SALKseq_087047 and LP4 and RP4 for SALKseq_123341. (B) Examination of transcript level of *MAPKKK18* in different *mapkkk18* mutants using semi-quantitative PCR (semi-qPCR). *ACT2* was amplified as a control. PCR was run for 25 or 30 cycles before the end products were examined through agarose gel electrophoresis. M and WT represent 1 kb DNA ladder and wild-type, respectively. (C) The representative plants of 26-d-old soil-grown *mapkkk18* mutants and WT treated with water or 100 and 200 μM ABA for 7 and 14 d. Bar, 2cm. (D) Quantitative comparison of chlorophyll levels in rosette leaves of different *mapkkk18* mutants and WT plants as shown in (C). Six independent plants for each genotype were assayed and three biological replicates were used. Data represents averages ± SD. Different letters indicate significant differences between *mapkkk18* mutants and WT (Col-0) using Duncan's multiple comparison test (P<0.05).

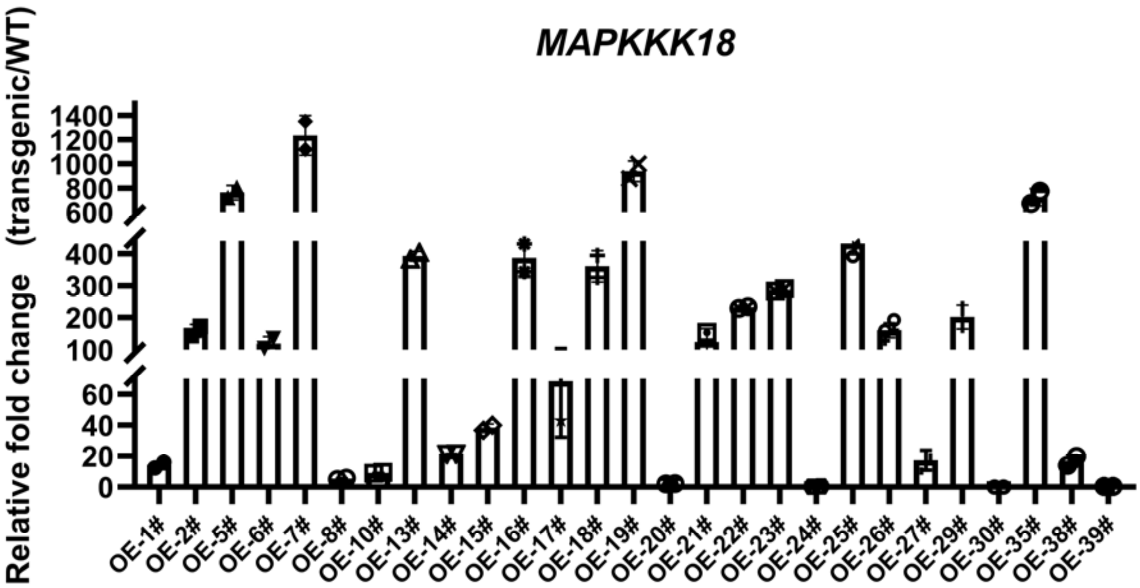

Figure S3. Examination of the *MAPKKK18* transcript level in different transgenic lines by qRT-PCR. Seven-d-old seedlings grown on 1/2 MS agar medium were collected for RNA extraction. qRT-PCR was performed to examine the expression level of *MAPKKK18* and the relative fold change was calculated compared to WT. *Polyubiquitin 10* (*UBQ10*) was used as an internal reference. Values are means ± SD of two independent replicates.

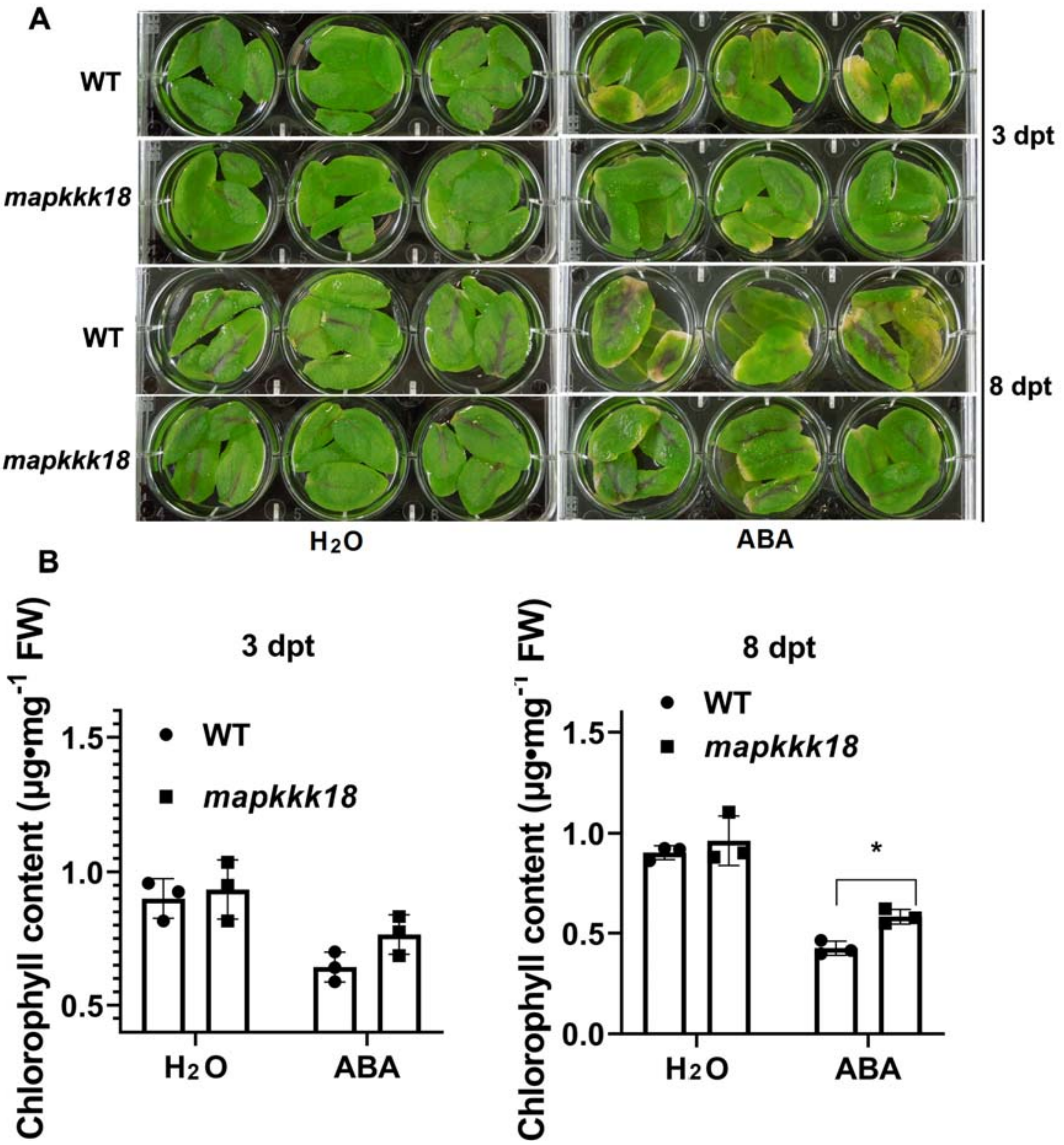

Figure S4. ABA-induced leaf senescence is delayed in *mapkkk18* mutant plants using detached leaf assay. (A) Phenotypes of the detached leaves of wild-type and *mapkkk18* mutant plants. The 6th and 7th leaves were obtained from 4-week-old soil-grown *mapkkk18* and WT plants. Detached leaves with abaxial side up were floated in H_2O or 25 µM ABA with a light intensity of 50 µmol m^{-2} s^{-1} for duration of 3 and 8 dpt (days post-treatment). (B) The chlorophyll content between *mapkkk18* and WT was compared. Each value represents the mean ± SD of three biological replicates. Asterisks denote significant difference by Student's *t*-test ($P<0.05$).

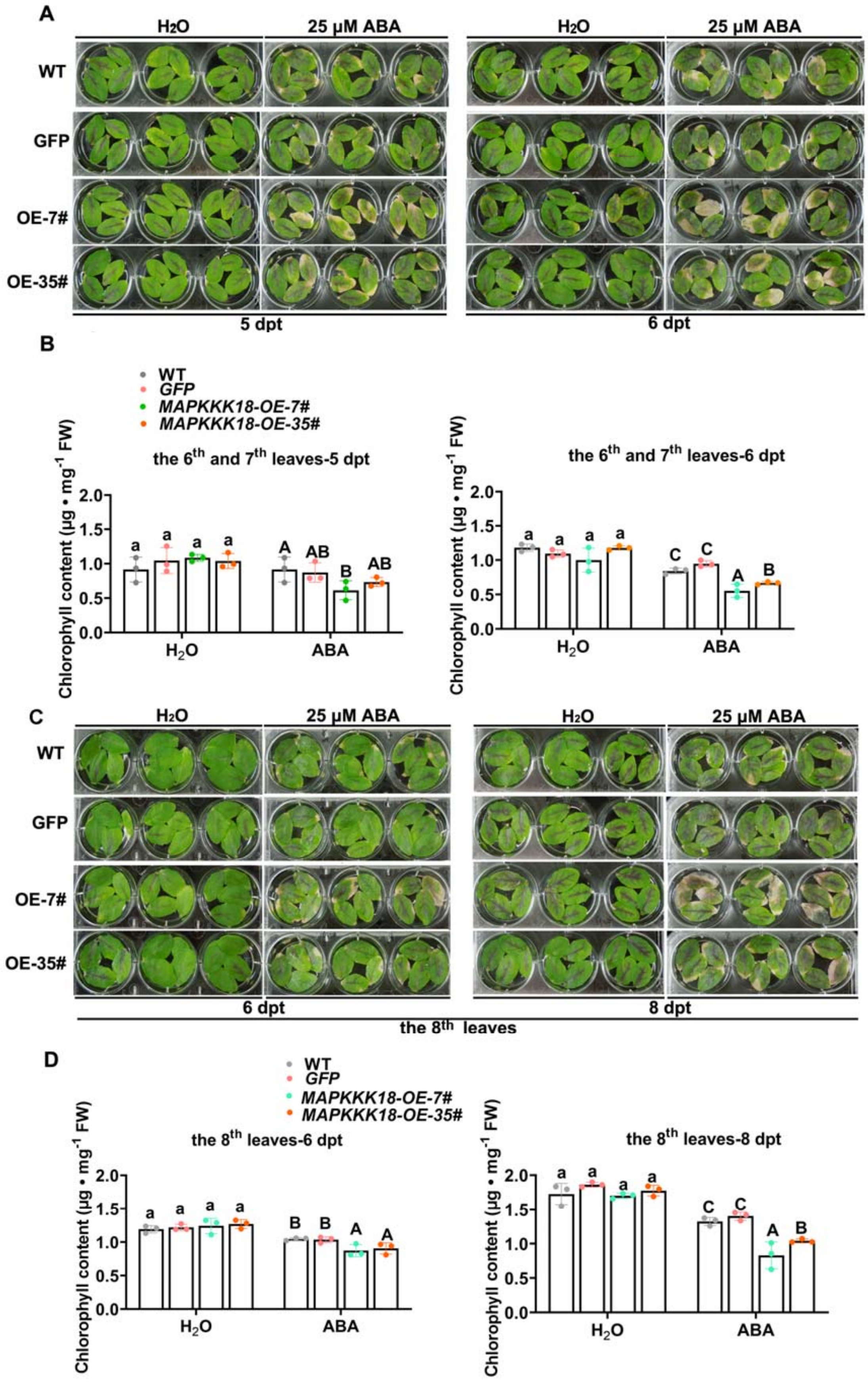
A
H2O
25 μM ABA
WT
GFP
OE-7#
OE-35#
5 dpt
6 dpt
B
WT
GFP
MAPKKK18-OE-7#
MAPKKK18-OE-35#
the 6th and 7th leaves-5 dpt
the 6th and 7th leaves-6 dpt
Chlorophyll content (μg • mg-1 FW)
H2O
ABA
C
the 8th leaves
D
the 8th leaves-6 dpt
the 8th leaves-8 dpt

Figure S5. ABA-induced leaf senescence is accelerated in *MAPKKK18* overexpression lines using detached leaf assay. (A) Representative images of the 6th and 7th leaves after treated with water or 25 μM ABA for 5 and 6 d. Leaves were cut from 4-week-old soil-grown WT, *GFP* transgenic control, *MAPKKK18*-OE-7#, 35# plants and were floated in H_2O or 25 μM ABA with abaxial side up for 5 or 6 d with a light intensity of 50 μmol m^{-2} s^{-1}. (B) The comparison of the chlorophyll content among *MAPKKK18* overexpression plants, *GFP* overexpression control, and WT plants as described in (A). (C) Representative images of the 8th leaves after treated with water or 25 μM ABA for 6 and 8 d. (D) The comparison of chlorophyll contents among *MAPKKK18-OE* plants, *GFP* overexpression control and WT as described in (C). In both (B) and (D), each value represents the mean ± SD of three biological replicates. Different letters indicate significant differences based on one-way ANOVA test followed by Duncan's multiple comparison test (P<0.05).

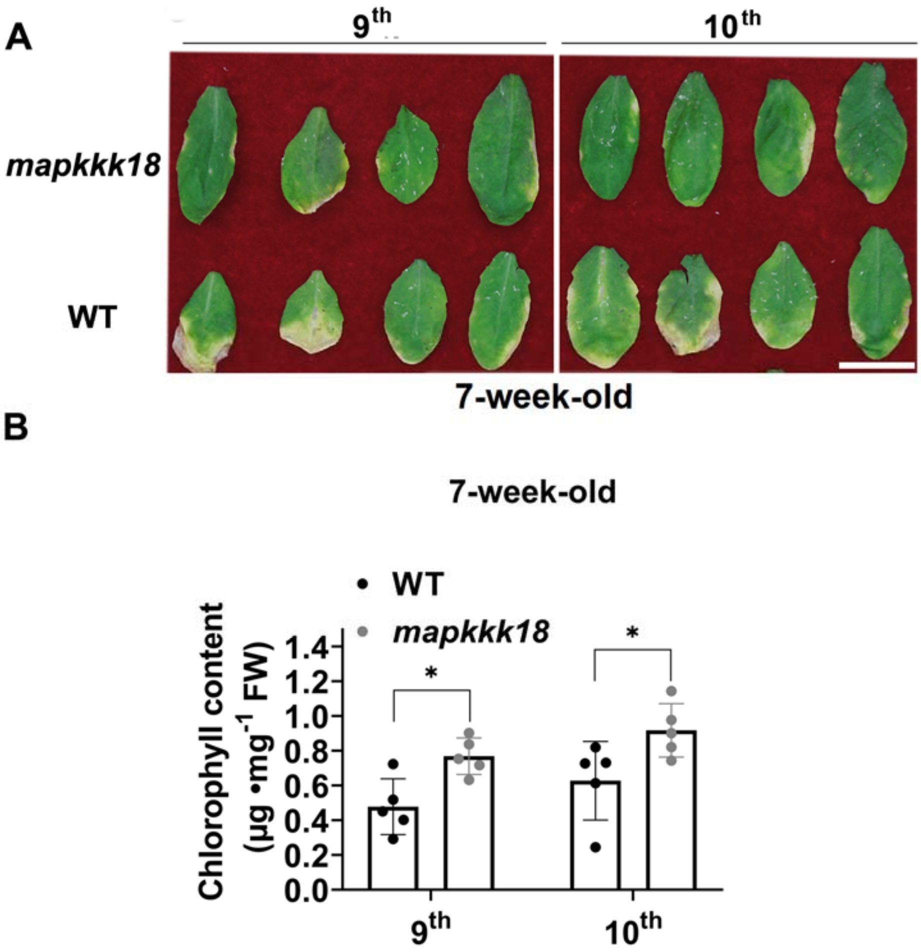

Figure S6. Age-dependent senescence is delayed in the *mapkkk18* mutant. (A) Representative leaf series from the 9th to 10th rosette leaves of *mapkkk18* and WT (Col-0) in 7-week-old plants were compared. Bar, 2 cm. (B) The chlorophyll contents between *mapkkk18* and WT were compared and asterisks denote significant differences by Student's *t*-test ($P<0.05$). Each value represents the mean ± SD of five biological replicates.

>*MAPKKK18* (At5g05120) genomic DNA sequence

ProMAPKKK18-ChIP-F3-F

>>>>>>>>>>>>>>>>>>>

gtgttggtaaacaacggtgaacgaactataacttgatagttacaaatgatatatttaaaagtagatatc

atagtgtttgtgataaaactttttttcaaaatgtaattcaaacaatttgaattgagtaggttgttgcttg

aagtaggcgttcctcattatacgaatgtaattcaaacaatttgaattgagtaggttgttgcttgaagta

<<<<<<<<<<<<<<

ProMAPKKK18-ChIP-F3-R

ggcg-ttcctcattatacgaat***cttttgcgtttctctttgttttcatgccacgtgttgtctctaccg***ct

cacacgccacaagaacattcttgaaaaggcttatcaaacaactttaagaacatatttctattctaccca

<<<<<<

Acagtaagaaaaataaaatatatcagttatttggtttgacatagctagcgtccaactaattagaaatga

tcagtttatttacattatcatccaagaaaatgcattgattcataatgattaatacaaattttcattggt

tattcatgggtactttttcttaccattaatgctaaaaaataaaataaaattcaccaatgtacaataagtt

tcttaattagcacactttttttgcgtttggtcatctgttttatgtttggttttataagtatagcattaca

attattattttagctttaaaaacttaaaatgtaaattttaaacacgtgaatgtgcaagttattatgcat

gtggttaataataaagggtacatgtgcgtatgagctgtgaatgtgcgtatatggcagtggacagccgta

aatgtctgaccaaaatcgttttccttatctttttacccaacagtactactactgccttaggttaaaact

catcttgtcggacgcttcttcttacaagtttacgtcttttatattctaaaattgtaacaatatattaag

tttgagaaatctttcttattaattattactacgtcatttttatgtgttaactatttttgtcgaagactt

ttttattcttgatcaattagtccgaaaatcactactaattcgtaaacaaatttttattattaaatacct

ctctgtttttcctcatttgagaacgtgatataaaatcaacataatcgaatcttcacagttcacactagt

ttatcattataataagtgaggggattacattttaggtatttagagaaaacggggaaaagataccaaaat

atcttagagacccgtggttctatatgtacattttaatttttaatttgctaaagtcagccatgaatacta

tagtatacctagtcagatttgatgatgatggttgttaaacccaccgctcctcctagtcaaacatcttct

acaatttccaaacacattcaaaaaacgcagaaaaaaaaaaaaaaaatgtgtatatgtgttacaattctt

aaaagaaaatgtaaagccagataattgttacaagagatgtacaaagctccaagtgtgaagaacatcaac

ProMAPKKK18-ChIP-F2-F

>>>>>>>>>>>>>>>>>>>

Atgaatatgatccatgaaatctaccaaatttccgtgcttgcactttcccatacatttttgtttttcacc

taactcttacgaacctaaaataaaataaaatacaaaaaaagggagaaagatttgagcacttgtcgacac

atcgtagcatcc***tacctctgccgacacgtggctcgagacaaat***acggaactcccggtcggaacagaaac

aaatacttattttgactacctgaaattcgacacgtcatcaccttctagactcaaaagagtacaaaggcc

<<<<<<<<<<<<<<<<<<<<

ProMAPKKK18-ChIP-F2-R >>>>>

aatgagactctaccattcctaacgaagtgtcacgtcacttcctatcaaacctttccttaaatacgttca

ProMAPKKK18-ChIP-F1-F

>>>>>>>>>>>>>>>>>>>

Gttcactgccgcgtcactcctctttttggtcacgtttcctatttttgtactaatacttcttcttctct

ctacacatattttgttggtaatcacatcgaagatt***cgaaattggatcacatttggtaatcaactttttt***

ttccaaagtctccacgtgtatggacgattacatacgtacacttatttattttcgctttggtggataaac

tcgatgttaccaaaagtatacactatctaattaccgaaccaaaattaatctgaaacccttctatatata

```
                                                         <<<<<<<<<<<<<<<<<<<<<<<<<<
                                                              ProMAPKKK18-ChIP-F1-R
tatactttctttgcaataacagagtgaaagcaaacattcaaaaaaaaaaaaaaaccatttttttttct
tactcaaatctttttagtatcattctccaaATGAATTGGACTAGAGGAAAAACTTTAGGCCGTGGCTC
AACGGCCACCGTTTCCGCCGCCACTTGTCACGAATCCGGCGAAACACTCGCCGTAAAATCCGCCGAGTT
TCACCGGTCGGAGTTCTTGCAAAGAGAAGCCAAGATTCTCTCCTCATTAAACTCTCCGTACGTCATCGG
ATACAGAGGATGCGAAATTACGAGAGAGCCCTTCCACAACAACGGAGAAGCTACGACTTACAGTCTTCT
TATGGAGTACGCGCCGTACGGAACGTTGACCGACGTAGCGACCAAGAACGGCGGCTTCATTGACGAGGC
TCGGGTCGTGAAGTACACGCGCCAGATACTTCTTGGGTTGGAGTACATTCATAACTCAAAGGGAATTGC
GCATTGCGACATTAAGGGAAGCAACGTGTTGGTCGGAGAGAACGGAGAAGCCAAGATCGCTGATTTCGG
GTGTGCGAAATGGGTTGAACCGGAAATAACCGAACCGGTTAGAGGAACGCCGGCGTTCATGGCTCCAGA
AGCGGCGCGTGGAGAGAGACAAGGGAAAGAGAGTGATATATGGGCGGTGGGTTGTACGGTGATAGAGAT
GGTTACCGGGTCTCAGCCGTGGATTGGGGCGGATTTTACCGACCCGGTTTCGGTTCTTTACCGGGTCGG
ATATTTAGGTGAGTTACCAGAGCTGCCTTGCTCGCTTACGGAACAAGCAAAGGATTTTTTAGGAAAGTG
TTTGAAAAAAGAAGCGACGGAGAGATGGACAGCGAGTCAACTACTAAACCATCCGTTCTTGGTTAATAA
AGAACCGGAATTGGTAACCGGTTTGGTTACGAACTCACCGACAAGTGTGACGGATCAAATGTTCTGGAG
ATCAGTGGAAGAGGAGGTGTCAGAGGACCGTTCAAGCTGGTGGGAATGTCACGAGGATGAGAGAATTGG
AGTGCTAAGCTGGATTGGTCATGTTGTGGTGGAGTCCACATGGGACTTGGACGGTGAGGATTGGATCAC
GGTTCGACGGAATTAGtcggttcacatgaaggagcgttgattattcgattcaattagtgaagatattgt
tttagtttagtagaagttgaggtttagagattttcatgtacatttcctaattcatcatttttttatcccc
acaaatccaatttttgttattcattgattatatgttataatatgagtcatgattgattatctaaaaaca
acaatttagttaaaacaattgtgaattggactttgatttcttaatctaatagaacaaatggacaagagc
cattgaaccaagtattgctagaatttgcaccaattatcttgaacacactagttagttcagttgcatgaa
gttagaggtagtcaagttaggtaaaagattgacaattatgataaaacatactaaagatggtttttttgtg
ggagtgtaatgattttaacttgtggggaaagtgagaaatgaaggcaacaaaaaggtgccgaatttgttt
gttggttggttaattggtcattttgagggtttaggcaaactaaaagattcgaaaggaatcttttggtaa
ggattcgcatttccatttagtttttttttgggtaatttggtacgcttcggttaggccatgttgatgag
tcattcattatgcctttgcttccacacaaatctctaattatcatatggtaaataattttaaaggacaaa
aggttaagacctaccttgttactacttaacctcaattcatcttccccttaatctattattcacaataat
catcaagaaacactttcttacaaggaagtaccaaatcatagatgtgtcatcatcatcaaaaaatctctg
aatgtaagaagttaaaaacgggcttaagtaacacataccattaccaccatactactacaccgagaccag
atcttcatggtcatacaaaagcgttttcatctgttagaaaaaagTTATGTTGTAAACAAGAACCTCATA
                                                         ProMAPKKK18-ChIP-CK-F
                                                         >>>>>>>>>>>>>>>>>>>
TCTTCCTCGGTCAGCTTTCCTATAGCCTCCTGAGAGCCACCAACCGTCctgtaatgaacaaaaatgtgt
ttataataaccttcaatagtagttttaaggttcggaaaagaagttgagaatgttttacttacCCTTCAA
ACACAAGTTCCTTCTTCTTCTGAAGCCTTAAAATCCTTTCCTCCACTGTGTTCTCTATTATGAATCTCA
CAA                                             <<<<<<<<<<<<<<<<<<<<
                                                 ProMAPKKK18-ChIP-CK-R
```

>*ACT7* (AT5G09810) promoter

```
                                                              AtACT7-ChIP-F
                                                             >>>>>>>>>>>>>>
Agaaaaaaataaaagagtgagaaaaatcgtagagctatatattcgcacatgtactcgtttcgctttcct
```

```
>>>>>>>>>>
Tagtgttagctgctgccgctgttgtttctcctccatttctctatctttctctctcgctgcttctcgaat
cttctgtatcatcttcttcttcttcaaggtgagtctctagatccgttcgcttgattttgctgctcgtta
                        <<<<<<<<<<<<<<<<<<<<<<<<
                              AtACT7-ChIP-R
Gtcgttattgttgattctctatgccgatttcgctagatctgtttagcatgcgttgtggttttatgagaa
aatctttgttttggggttgcttgttatgtgattcgatccgtgcttgttggatcgatctgagctaattc
ttaaggtttatgtgttagatctatggagtttgaggattcttctcgcttctgtcgatctctcgctgttat
ttttgtttttttcagtgaagtgaagttgtttagttcgaaatgacttcgtgtatgctcgattgatctggt
tttaatcttcgatctgttaggtgttgatgtttacaagtgaattctagtgttttctctttgagatctgtg
aagtttgaacctagttttctcaataatcaacatatgaagcgatgtttgagtttcaataaacgctgctaa
tcttcgaaactaagttgtgatctgattcgtgtttacttcatgagcttatccaattcatttcggtttcat
tttactttttttttagtgaaaaATG
```

Figure S7. Analysis of *MAPKKK18* promoter and relevant primer design.

Lowercase and upercase letters represent promoter region (including 5’untranslated region, UTR) and coding region, respectively. The letters highlighted in red are 5’UTR and 3’ UTR. The letters highlighted in italic and underlined are regions for designing probes for EMSA. Of them, probes 2 and 3 are directly synthesized while probe 1 is amplified by primers highlighted also in grey color. The ABRE (Abscisic Acid-Responsive Element) *cis*-elements were highlighted in green. The fragments used for EMSA were P1, P2 and P3 for the *MAPKKK18* promoter (underline). The sequences highlighted in grey background were primer sequences. qPCR with primers specific to the promoter of the *ACT7* gene was used as a non-binding control.

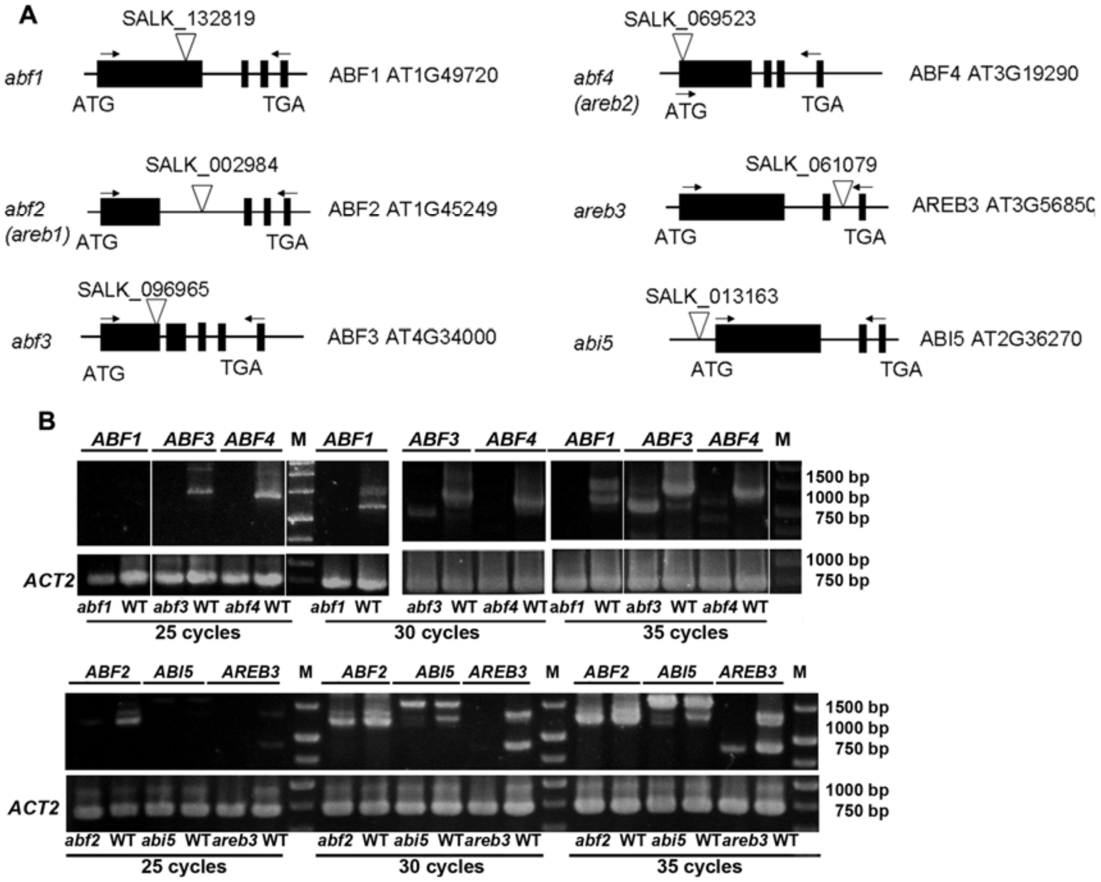

Figure S8. The identification of *abf* mutants. (A) The insertion sites of T-DNA in sequences of genomic DNA of *abf* mutants (*abf1,* SALK_132819; *abf2,* SALK_002984; *abf3,* SALK_096965; *abf4,* SALK_069523; *areb3,* SALK_061079; *abi5,* SALK_013163). Triangles indicate T-DNA insertions, black rectangles represent exons, and black lines represent introns and untranslated region (UTR). Arrows stand for locations of primers used for semi-quantitative RT-PCR (semi-qRT-PCR). (B) The expression analysis of *ABF* genes in *abf* mutants through semi-qRT-PCR. *ACT2* was used as an internal control. PCR was run for 25, 30 or 35 cycles before the end products were subjected to agarose gel electrophoresis. M stands for 1 kb DNA ladder.

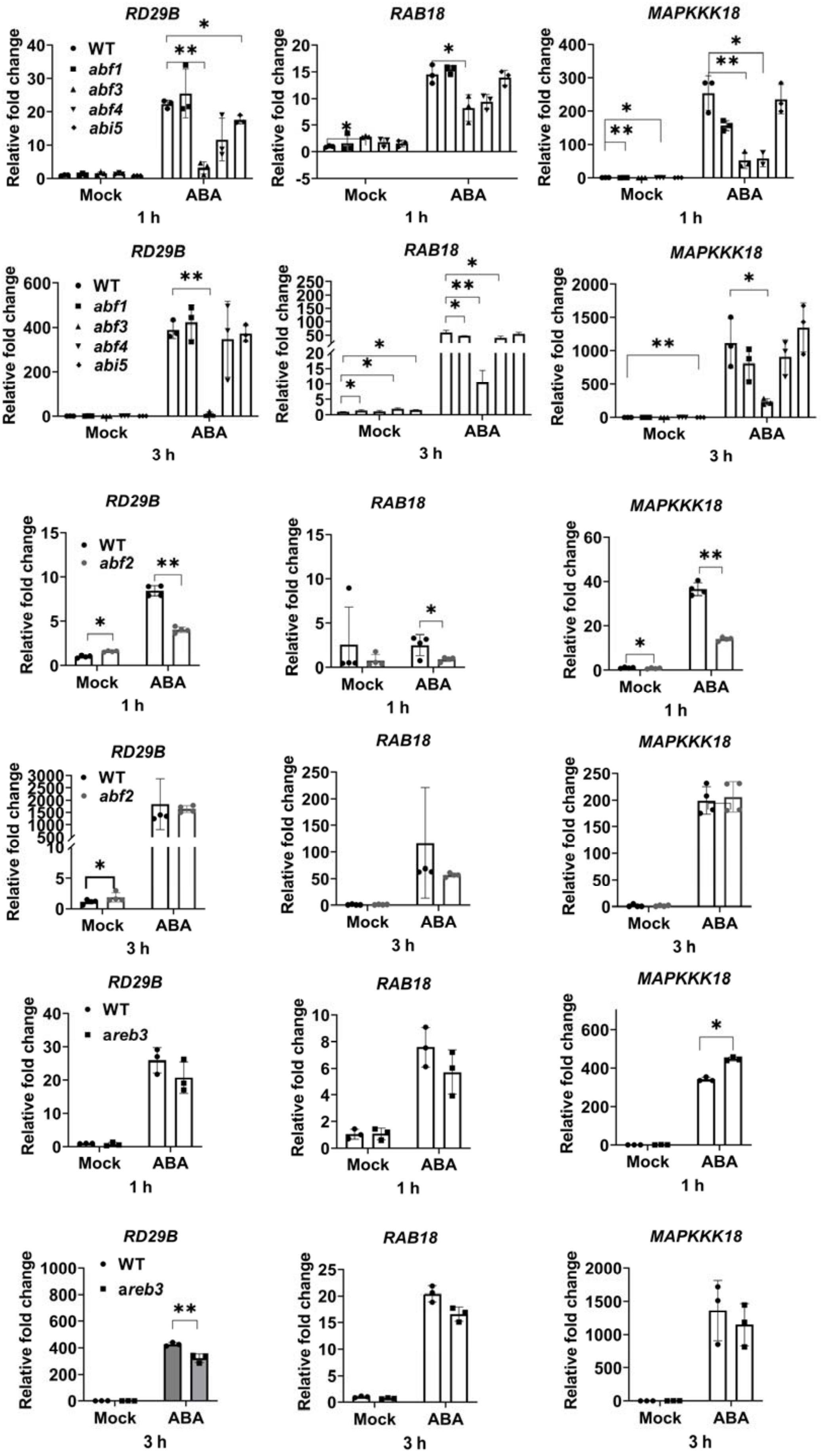
RD29B
RAB18
MAPKKK18
WT
abf1
abf3
abf4
abi5
abf2
areb3
Relative fold change
Mock
ABA
1 h
3 h

Figure S9. Analysis of *MAPKKK18* expression in *abf1*, *abf2*, *abf3*, *abf4*, *abi5, areb3* single mutants, and WT after ABA treatment. (A) Analysis of the expression level of *MAPKKK18* in *abf1*, *abf2*, *abf3*, *abf4*, *abi5*, *areb3* single mutants, and WT after 50 μM ABA treatment for 1 h and 3 h through qRT-PCR. The fold change of each gene was calculated by dividing the expression level in the WT plants under mock treatment. Data are means of at least three independent biological replicates ±SD. Asterisks indicate significant differences between WT and mutants by Student's *t* test (*, $P < 0.05$; ** $P < 0.01$).

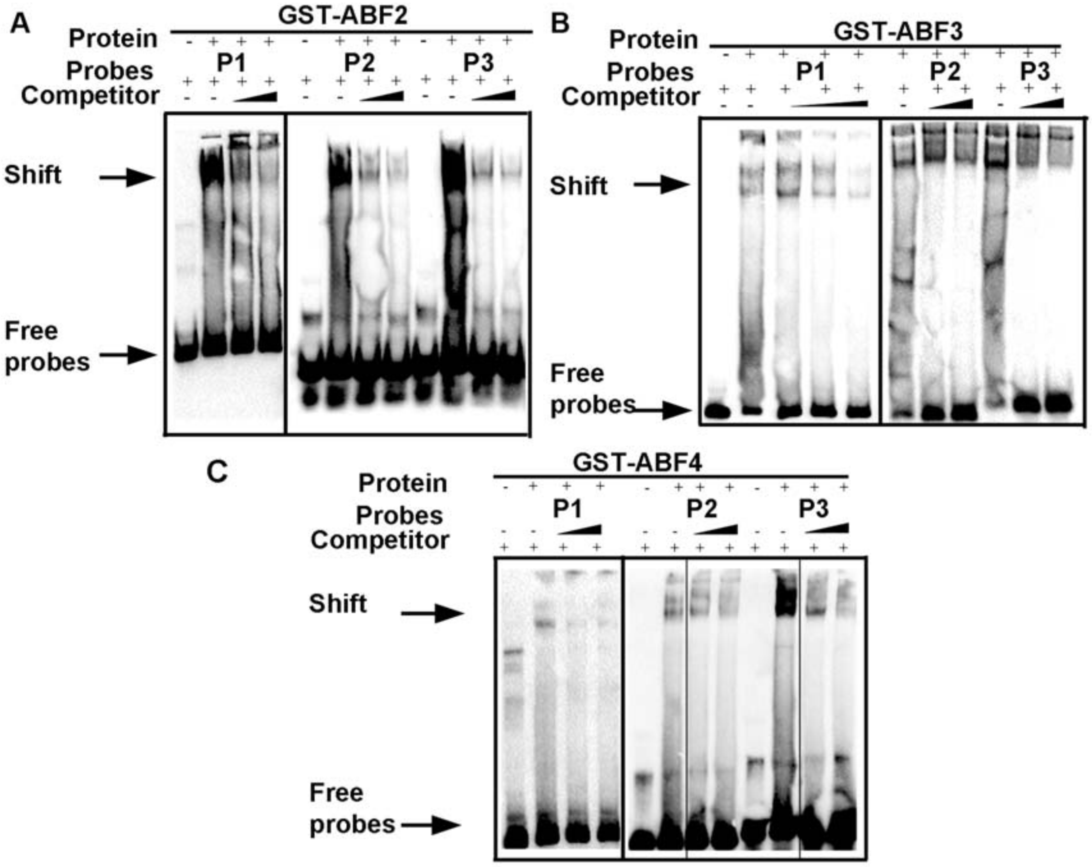

Figure S10. ABFs physically bind to the promoter region of *MAPKKK18*. (A-C) Electrophoresis mobility shift assay (EMSA) of binding of recombinant GST-ABF2 (A), GST-ABF3 (B) and GST-ABF4 (C) to ABRE-containing promoter regions of *MAPKKK18* as indicated by P1, P2, and P3. Competitive probes were added in a molar excess as shown above the lanes. The bands of protein-DNA shift and free probes were indicated by arrows at the upper and lower parts of each image, respectively. "+" and "-" stand for the presence and absence of corresponding

components in each binding reaction, respectively.

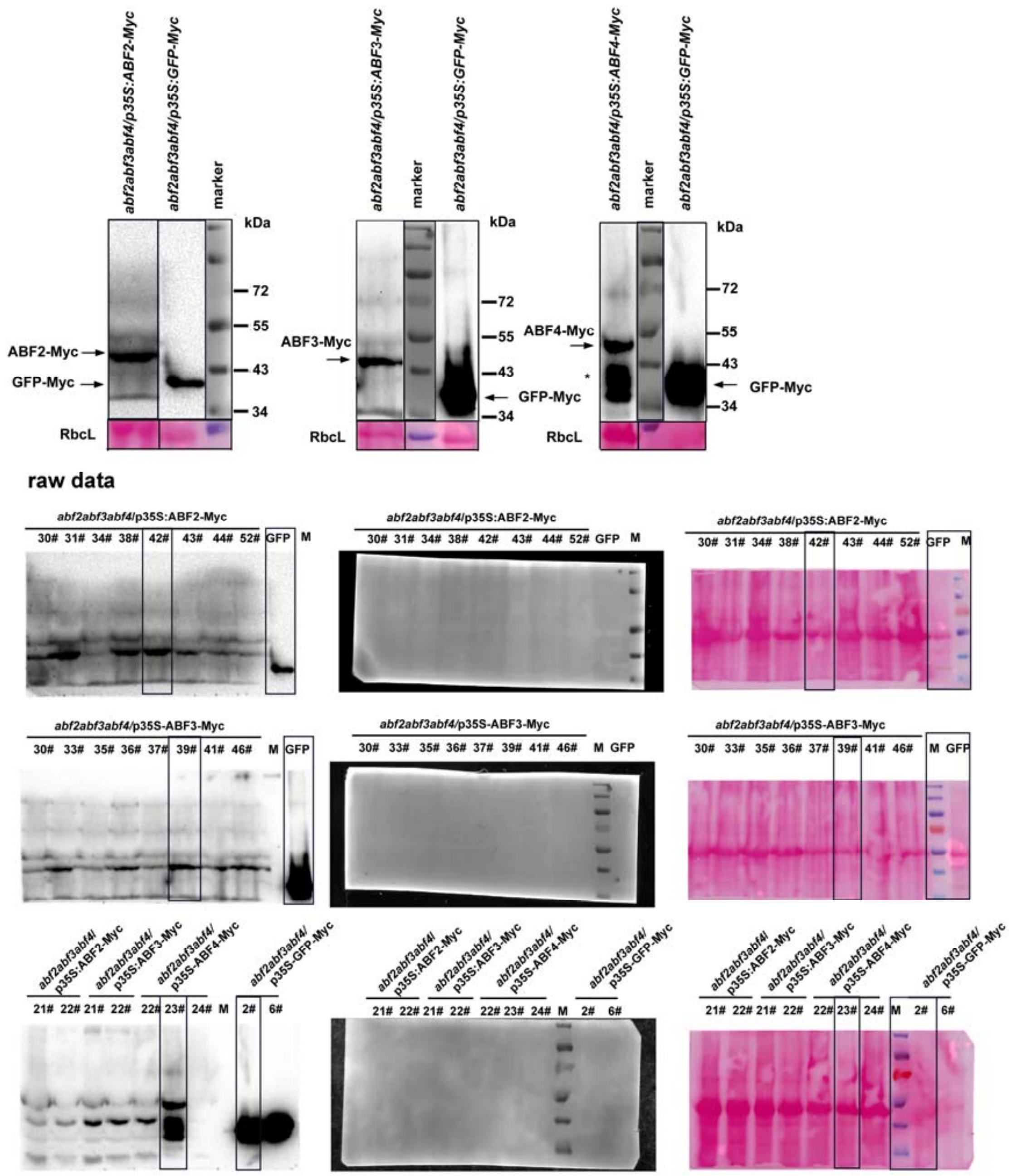

Figure S11. Immunoblot detection of ABF2, ABF3, and ABF4 in transgenic Arabidopsis overexpressing *ABF2*, *ABF3*, and *ABF4*.

Protein extracts were prepared from transgenic Arabidopsis leaves after treated with 50 μM ABA and 10 μM MG132 for 12 h. Anti-Myc antibody was used to detect ABFs-Myc. The abundant large subunit of Rubisco (RbcL) was stained with Ponceau S staining to verify equal loading. The asterisk indicates a non-specific band. The raw

images were shown in the lower panel with red rectangles indicating cropped areas.

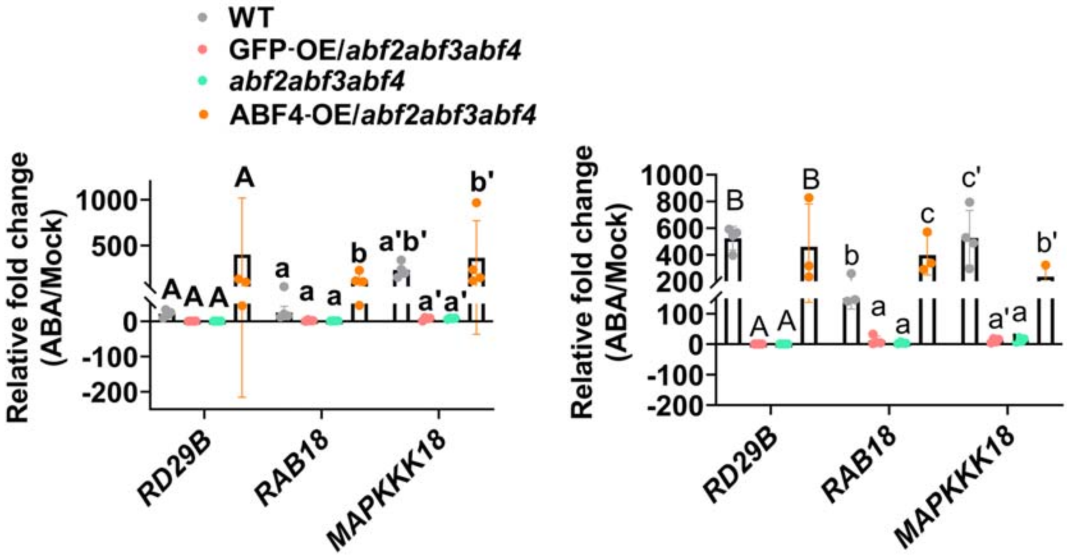

Figure S12 *MAPKKK18* expression is recovered in *ABF4-OE*/*abf2abf3abf4* transgenic lines. (A) Analysis of the expression level of *MAPKKK18* in WT, *abf2abf3abf4* triple mutant, control *GFP*-OE/*abf2abf3abf4* and *ABF4*-OE/*abf2abf3abf4* after 50 μM ABA treatment for 1 h (left) and 3 h (right). The fold change of each gene was calculated by dividing the expression level under mock treatment. Each bar represents the mean ± SD of four biological replicates. Different letters indicate significant differences by one-way ANOVA test followed by Duncan's multiple comparison test ($P < 0.05$).

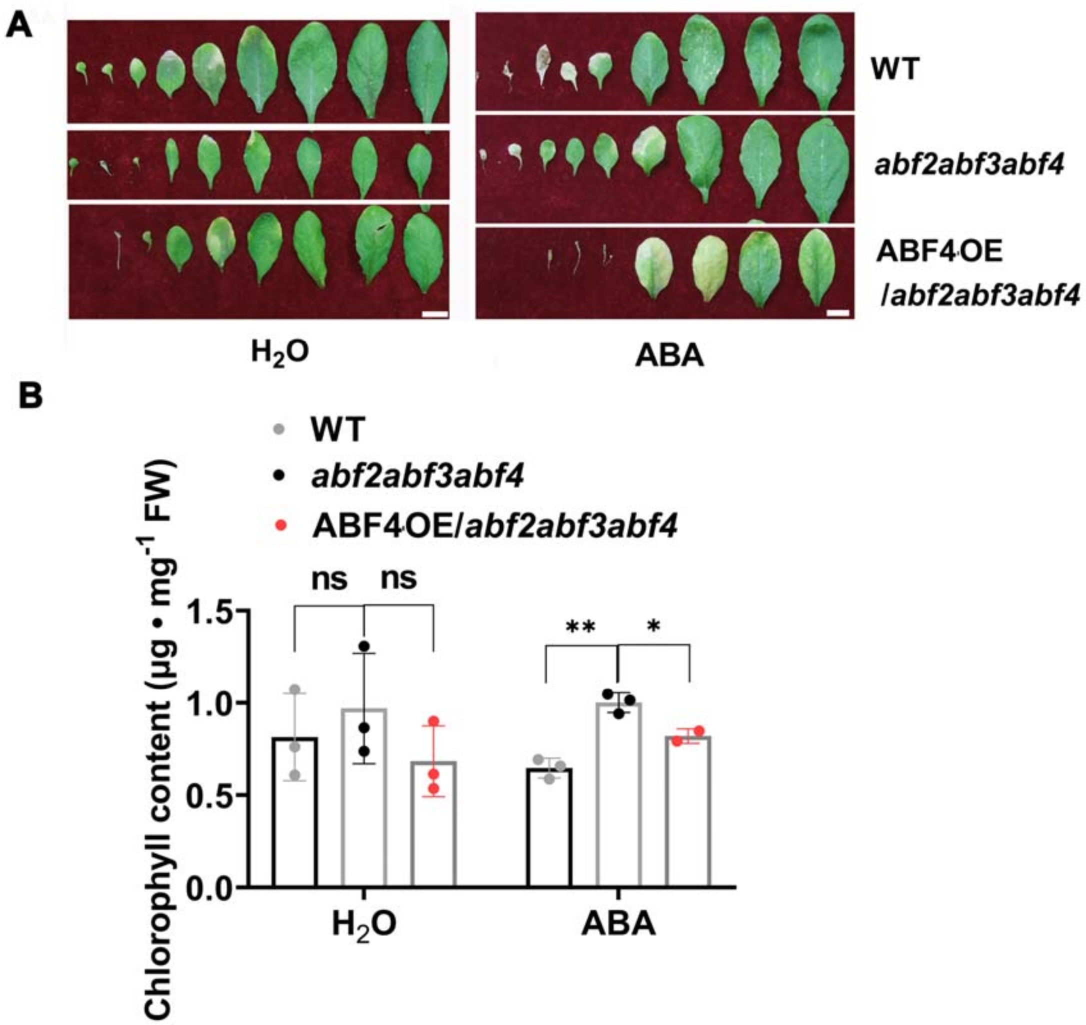

Figure S13. ABA-induced leaf senescence is accelerated in *ABF4-OE/abf2abf3abf4* line. (A) Representative images of the 5th leaves after treated with water or 200 µM ABA for 10 d. Rosette leaves of 26-d-old soil-grown WT, *abf2abf3abf4*, and *ABF4-OE/abf2abf3abf4* were sprayed with H_2O or 200 µM ABA daily. Images were photographed at 10 days post-treatment. Scale bar, 1 cm. (B) The comparison of the chlorophyll content among WT, *abf2abf3abf4*, and *ABF4-OE/abf2abf3abf4* plants as described in (A). Each value represents the mean ± SD of three biological replicates. Asterisks indicate significant differences between WT and mutants by Student's *t* test (*, $P < 0.05$; ** $P < 0.01$).

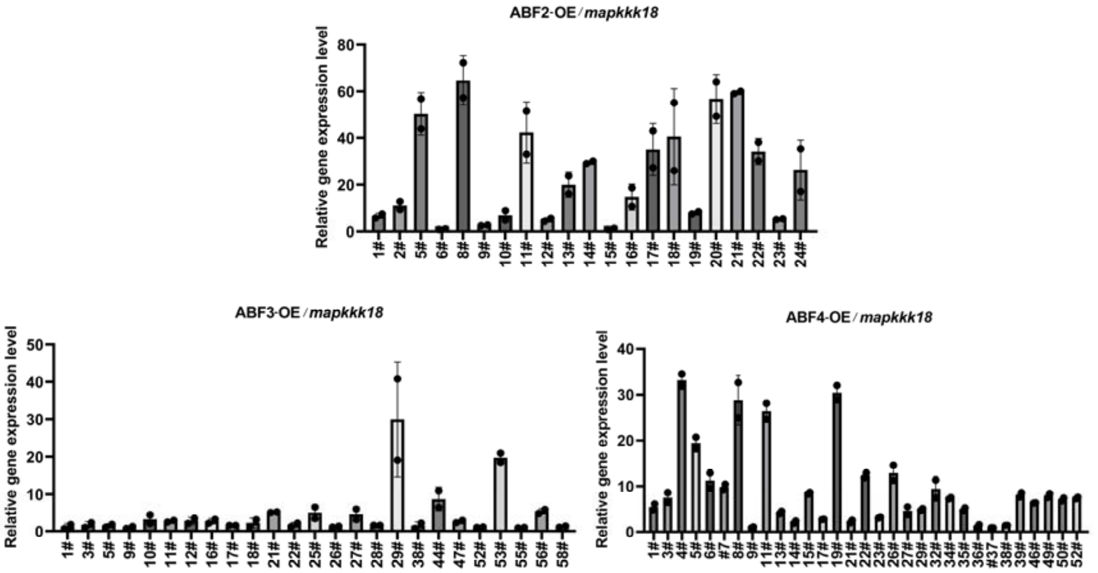

Figure S14. Expression levels of ABF genes in transgenic lines overexpressing *ABF2*, *ABF3*, and *ABF4* in the background of *mapkkk18* mutant by qRT-PCR. Seven-d-old seedlings grown on 1/2 MS agar medium were collected for RNA extraction. qRT-PCR was performed to examine the expression level of *ABFs* and the relative fold change was calculated compared to that in WT. *Polyubiquitin 10* (*UBQ10*) was used as an internal reference. Values are means ± SD of two replicates.

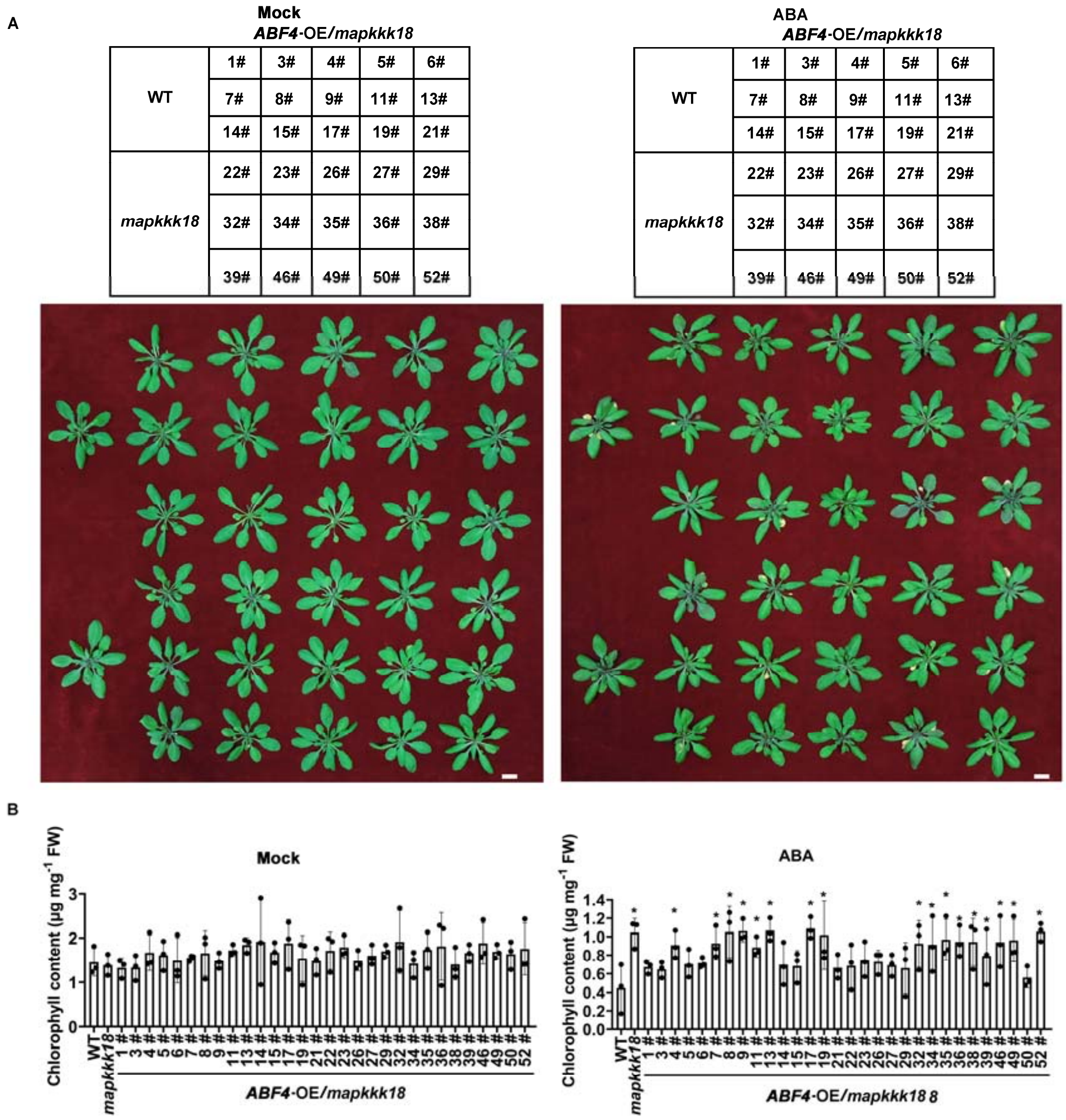

Figure S15. The earlier senescence of *ABF4* overexpression lines is compromised in the *mapkkk18* background after 50 µM ABA treatment. (A) 30 independent transgenic lines of *ABF4-OE/mapkkk18,* together with WT (Col-0) and *mapkkk18* mutant were grown in soil. Plants were then sprayed with 50 µM ABA daily for 8 d before photographed. Scale bar, 1 cm. (B) The comparison of chlorophyll contents of rosette leaves in plants as shown in (A). Each value represents the mean ± SD of three biological replicates. Asterisks indicate significant differences compared to WT by Student's *t*-test (* P<0.05).

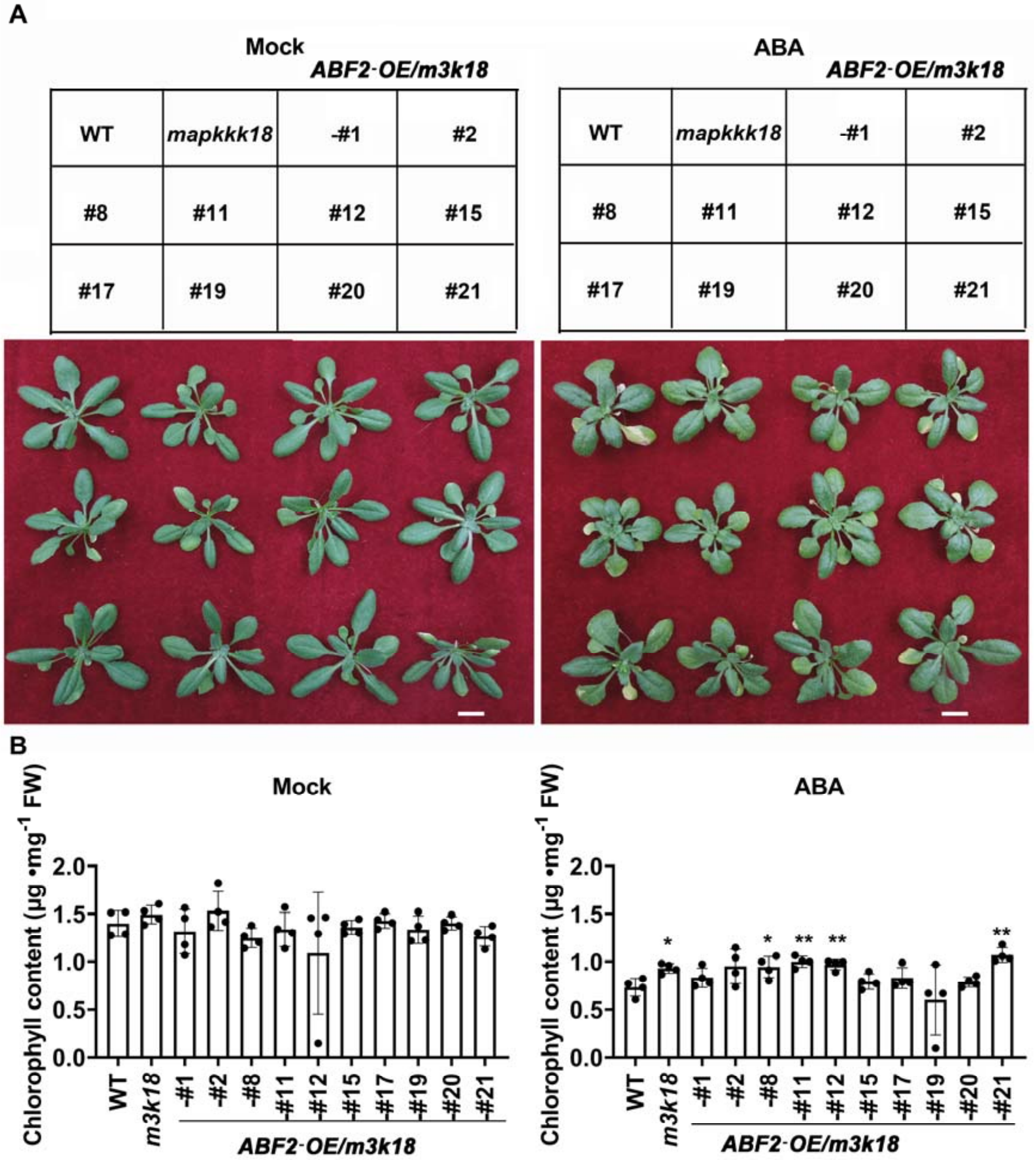

Figure S16. The earlier senescence of *ABF2* overexpression lines is compromised in the *mapkkk18* background after 50 μM ABA treatment. (A) 10 independent transgenic lines of *ABF2-OE*/*mapkkk18,* together with WT (Col-0) and *mapkkk18* mutant were grown. Plants were then sprayed with 50 μM ABA daily for 5 d before photographed. Bar, 1 cm. (B) The comparison of chlorophyll contents of rosette leaves in plants as shown in (A). Each value represents the mean ± SD of four biological replicates. Asterisks indicate significant differences compared to WT by Student's *t*-test (* P<0.05; ** P < 0.01).

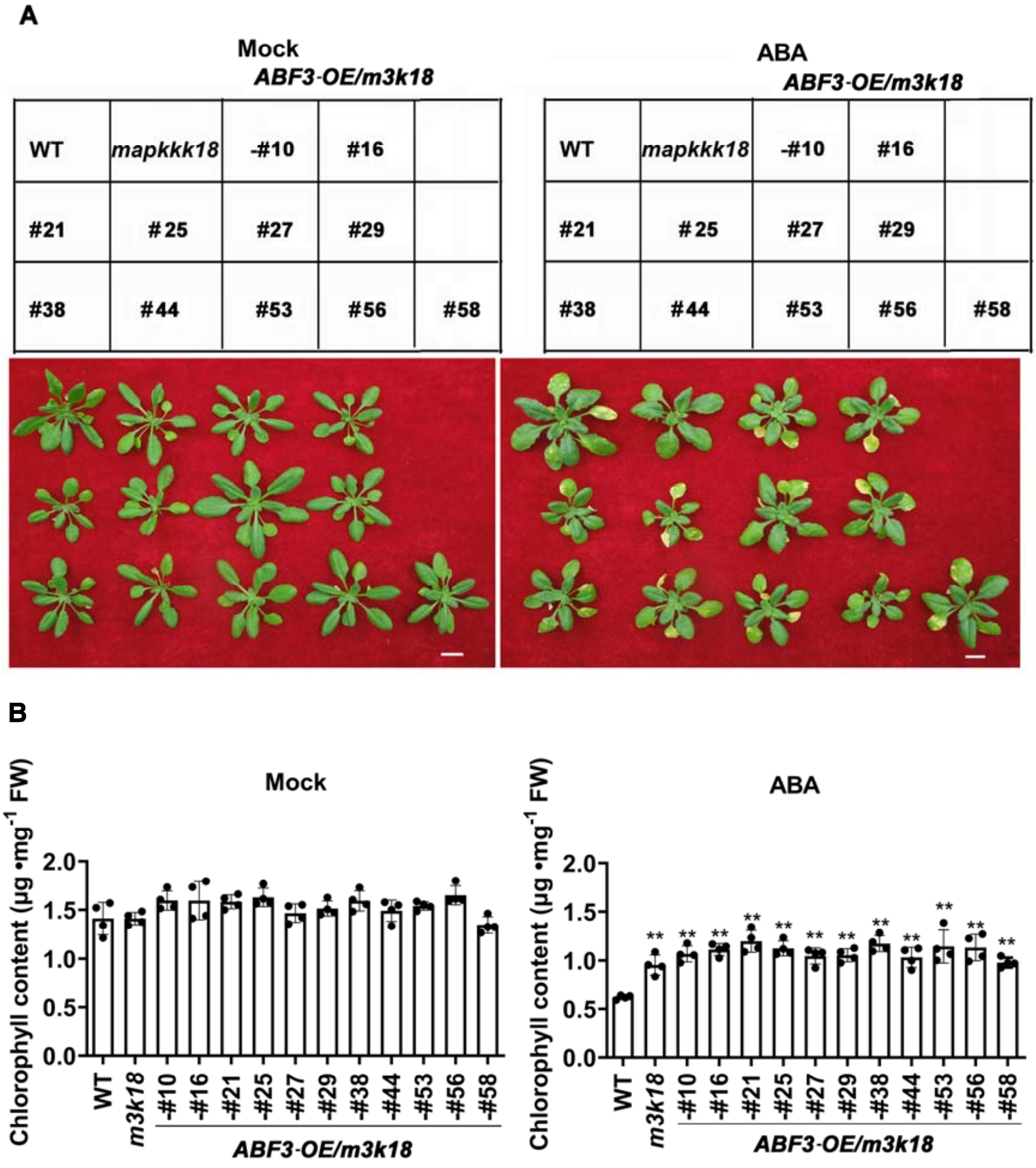

Figure S17. The earlier senescence of *ABF3* overexpression lines is compromised in the *mapkkk18* background after 50 µM ABA treatment. (A) 11 independent transgenic lines of *ABF3-OE*/*mapkkk18,* together with WT (Col-0) and *mapkkk18* mutant were grown. Plants were then sprayed with 50 µM ABA daily for 5 d before photographed. Bar, 1 cm. (B) The comparison of chlorophyll contents of rosette leaves in plants as shown in (A). Each value represents the mean ± SD of four biological replicates. Asterisks indicate significant differences compared to WT by Student's *t*-test (* $P<0.05$; ** $P < 0.01$).

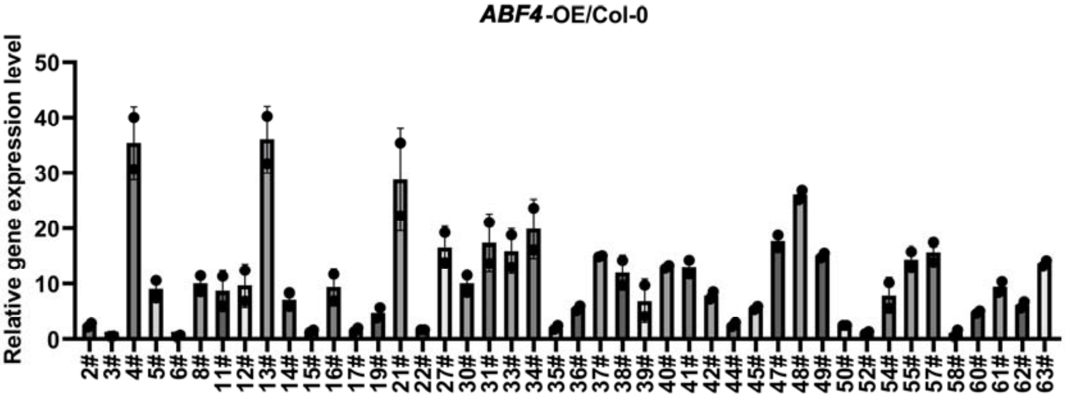

Figure S18. Analysis of expression level of *ABF4* gene in different overexpression transgenic lines in the background of Col-0 by qRT-PCR. Seven-d-old seedlings grown on 1/2 MS agar medium were collected for RNA extraction. qRT-PCR was performed to examine the expression level of *ABF4* and the relative fold change was calculated compared to that in WT. *Polyubiquitin 10* (*UBQ10*) was used as an internal reference. Values are means ± SD of two replicates.

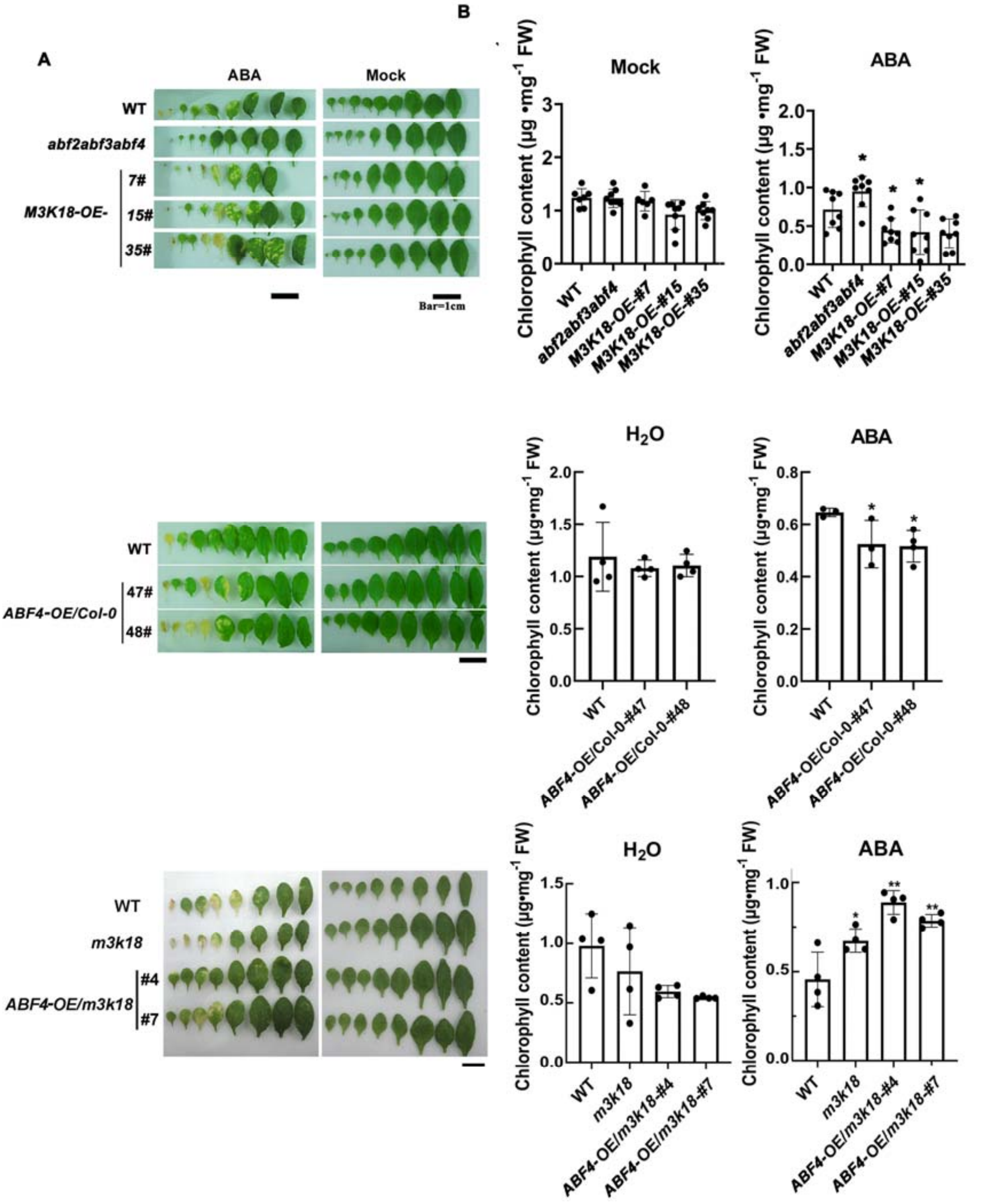

Figure S19. The earlier senescence caused by *ABF4* overexpression is abolished in the *mapkkk18* background. (A) Comparison of ABA-induced leaf senescence among different genotypes including WT, *mapkkk18*, *abf2abf3abf4, GFP/abf2abf3abf4, MAPKKK18*-OE, *ABF4*-OE/Col-0, and *ABF4*-OE/*mapkkk18* at 26-d-old. Plants were sprayed with 50 μM ABA (Coolaber) daily for 5 d before photographed. (B) The comparison of chlorophyll contents of the 5th and 6th true rosette leaves in plants as shown in (A). Each value represents the mean ± SD of at least four biological replicates. Asterisks indicate significant differences compared to WT by Student's

t-test (* *P*<0.05; ** *P* < 0.01).

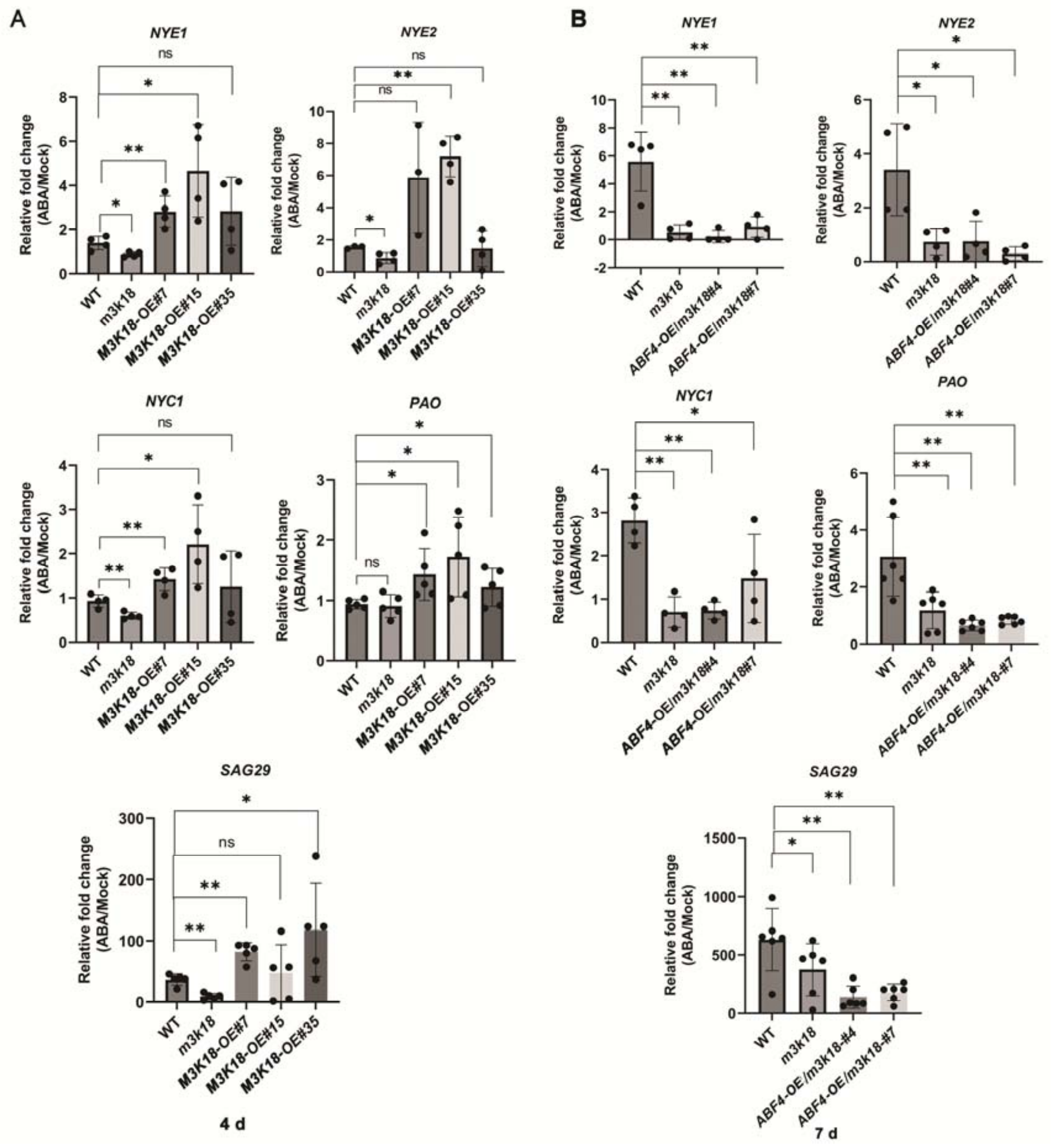

Figure S20. Expression analysis of *NYE1*, *NYE2*, *NYC1*, *PAO*, and *SAG29* genes in mutant and overexpression lines of *MAPKKK18* as well as in *ABF4-OE/m3k18* lines. 26-d-old WT, *mapkkk18* mutant, *MAPKKK18*OE (three lines, #7, #15 and #35), and *ABF4-OE*/*mapkkk18* (two lines, #4 and #7) lines were sprayed with or without 50 μM ABA daily for a total of 4 d or 7 d. The fold change of each gene was calculated by dividing the expression level under ABA treatment by mock treatment (distilled water). Each value represents the mean ± SD of four biological replicates. Asterisks indicate significant differences compared to WT by Student's *t*-test (* *P*<0.05; ***P*<0.01).

Supporting Experimental procedures

Construction of plasmids and generation of transgenic plants

For overexpression of *MAPKKK18*, the coding region of *MAPKKK18* was cloned to the pYWLHA vector (modified from pCsGFPBT, GenBank acc. No. DQ370426) between *Nco* I and *Sal* I sites under the control of cauliflower mosaic virus (CaMV) *35S* promoter with a C-terminal 2xHA tag. Similarly, the coding regions of *ABF2*, *-3*, *-4* were cloned to the p35SFC vector (modified from pCsGFPBT) under the control of CaMV*35S* promoter with C-terminal 3xFLAG tag.

The above constructs were introduced to wild-type (WT) or *mapkkk18* mutant Arabidopsis through agrobacterium-mediated floral dip method (58). Transgenic lines were selected on 1/2 x MS medium containing 25 mg L^{-1} hygromycin (Roche, Switzerland) for two generations to harvest homozygous T_3 seeds. T3 generation seedlings were screened through quantitative RT-PCR (qRT-PCR) to identify high expression lines. For the phenotypic assay, seeds of the different transgenic lines were planted and harvested under the same growth conditions simultaneously.

For ChIP-qPCR, plant materials were generated, in which *ABFs* tagged with 3xMyc were driven under the CaMV*35S* promoter. Basically, the coding regions of *ABF2*, *ABF3*, and *ABF4* were individually fused downstream of 3xMyc in the pFXMyc vector (modified from pCsGFPBT) using primers listed in Table S1, before transformed into *abf2abf3abf4* Arabidopsis plants. Homozygous T2 transgenic *3xMyc-ABFs* overexpression lines were identified through immunoblotting by Myc-tag-specific antibody (Cata #MA1980, Invitrogen).

Prokaryotic expression and affinity purification

The coding regions of *ABF2*, *ABF3,* and *ABF4* were individually subcloned into the pGEX-4T-1 vector (Amersham) downstream the *glutathione S-transferase* (*GST*) tag for prokaryotic expression. Afterwards, pGEX-4T-1-*ABFs* were transformed into Rossetta-gami (DE3) pLysS or BL21 –Codonplus (DE3) strain (Stratagene, USA) for protein induction by isopropyl thio-β-D-galactoside (IPTG) for 6 h at 25 ℃. Cell pellets were then collected for lysing, and fusion proteins were purified using

GST-bind resin (Novagen, USA).

Subcellular localization assay

The coding sequence of *MAPKKK18* was subcloned into a modified pYJGFP vector through *Nco* I/*Sal* I sites with C-terminal fusion with *GFP*. pYJGFP empty vector was used as the control. The suspension of *Agrobacteria tumefaciens* strain GV3101 harboring the above two constructs was individually infiltrated, together with the NLS-mCherry nuclear marker, into the abaxial surface of 21-d-old *N. benthamiana* leaves through 1-mL needleless syringes (53). 48 h later, leaf discs were checked on the FV1000MPE confocal microscope (Olympus, Japan).

Semi-qRT-PCR

The coding regions of different genes were amplified with primers list in Table S1. *ACT2* was used as an internal control. The program used for semi-qRT-PCR was as follows: initial denaturation at 94°C for 3 min, followed by 25 or 30 or 35 cycles of denaturation at 94°C for 30 s, annealing at 50 °C for 30 s and extension at 72°C for 30 s.

ABA-induced leaf senescence assay

ABA-induced leaf senescence was performed according to a previous report (6). The 6th, 7^{h} and 8th rosette leaves from ~26-d-old soil grown Arabidopsis plants were excised and floated with abaxial side up in a 5 mL of distilled water (control) or water supplemented with 25 μM ABA (Sigma-Aldrich, USA) in a six-well cell culture plate (Nest, China). Plates were then kept at 22 ℃ under a dim light (50 μmol $m^{-2}s^{-1}$). Photos were taken at two different time points as specified in the main text. The content of chlorophyll of detached rosette leaves was determined as described previously (55).

Natural senescence assay was conducted as described before (53). The 9th and 10th rosette leaves from 7-week-old of *mapkkk18* and WT Arabidopsis plants were evaluated.

Immunoblotting assay

Protein extracts were prepared from transgenic Arabidopsis leaves after treated with 50 μM ABA and 10 μM MG132 for 12 h. Total protein is extracted using an

extraction buffer (50 mM Tris-HCl, pH 7.5, 150 mM NaCl, 0.1% NP-40, 4 M Urea, 1 mM PMSF) (59). Proteins were resolved in 10% SDS-PAGE. Proteins were then transferred onto activated PVDF membrane through wet-transferring apparatus in TBS buffer (10 mM Tris, pH7.6, 150 mM NaCl) containing 1.0% Tween-20. Membrane was then blocked with 5% non-fat milk in TBST buffer. The membrane containing protein was then incubated with anti-Myc antibody (Invitrogen) in 1:5,000. Secondary antibody of goat anti-mouse IgG HRP secondary antibody (Pierce, 1:10,000) was applied for membranes blotted with Myc-tagged ABFs protein. Protein bands were developed by the SuperSignal West Femto Max Sensitivity Substrate kit (Pierce) on the ChemiDoc XRS+ system (Bio-Rad).

To examine the protein of MAPKKK18-GFP in different cellular fractions, tobacco leaves expressing *MAPKKK18-GFP* and GFP (control) was used to isolate proteins following the protocol as described before (60). Basically, 0.2 gram of tissue was ground in 0.4 mL of extraction buffer 1 (EB1) containing 10 mM Tris–HCl, pH 8.0, 0.4 M sucrose, 5 mM β-mercaptoethanol and 0.1 mM PMSF. Large tissue pieces were removed after the homogenate was filtered through the miracloth (Calbiochem). A volume of 75 μL was aliquoted and saved as 'total protein'. The crude nuclei were centrifuged at 2,500 x*g* for 20 min at 4 ◦C and the supernatant was saved and this is 'nuclear-depleted' protein. The pellet was resuspended in extraction buffer 2 (EB2) containing 10 mM Tris–HCl, pH 8.0, 0.25 M sucrose, 10 mM $MgCl_2$, 5 mM β-mercaptoethanol, 0.1 mM PMSF, 1% Triton X-100 plus 100 μL of 1xSDS-PAGE sample buffer. After the tube was sit on ice for 10 min, it was centrifuged at 15,000 x*g* for 10 min at 4◦C. After the supernatant was discarded, the pellet was resuspended in 0.4 mL of extraction buffer 3 (EB3) containing 10 mM Tris-HCl, pH 8.0, 2 mM $MgCl_2$, 1.7 M sucrose, 5 mM β-mercaptoethanol, 0.1 mM PMSF and 0.15% Triton X-100. Then, the tube was centrifuged at top speed at 4◦C for 60 min. The pellet was resuspended in 0.2 mL of M3 buffer supplemented with 10 mM potassium phosphate, pH 7.0, 10 mM β-mercaptoethanol, 0.1 M NaCl and 0.1 mM PMSF. After centrifuged at top speed for 5 min at 4◦C, the pellet was resuspended in 0.2 mL of sonication buffer containing 10 mM potassium phosphate, pH 7.0, 10 mM EDTA, 0.5% sarkosyl,

0.1M NaCl and 0.1 mM PMSF. A volume of 50 μL out of the mixture was used as 'nuclear' protein. Western blotting was performed using anti-GFP antibody (Invitrogen) as the primary antibody (1: 5,000) with the detection of signals carried out as described previously.

Accession numbers

Sequence data from this study can be identified in the TAIR database with Arabidopsis Genome Initiative (AGI) codes: *MAPKKK18*, AT1G05100; *ABF1*, AT1G49720; *ABF2*, AT1G45249; *ABF3*, AT4G34000; *ABF4*, AT3G19290; *AREB3*, AT3G56850; *ABI5*, AT2G36270; *ACT2*, AT3G18780; *ACT7*, AT5G09810; *RD29B*, AT5G52300; *RAB18*, AT1G43890; *NYE1(SGR1)*, AT4G22920; *NYE2* (*SGR2*), AT4G11910; *NYC1*, AT4G13250; *PAO*, AT3G44880; *SAG29 (SWEET15)*, AT5G13170.

Table S1 Primers used in this study

Primer name	Primer sequence (5'→3')	Purpose
MAPKKK18-NcoI-F	CGCCATGgacAATTGGACTAGAGGAAAA ACTTTAGGC	subcellular localization assay, constitutive overexpression in Arabidopsis, semi-qRT-PCR
MAPKKK18-SalI-R	CGCGTCGACATTCCGTCGAACCGTGATC C	subcellular localization assay, constitutive overexpression in Arabidopsis, semi-qRT-PCR
MAPKKK18-q-F	TGGATTGGGGCGGATTTTACC	qRT-PCR
MAPKKK18-q-R	CCTCTGACACCTCCTCTTCCACTGA	qRT-PCR
ProMAPKKK18-HindIII-F	CCCAAGCTTGTAATTCAAACAATTTGAA TTGAGTAGGTTGTTGC	Dual-LUC
ProMAPKKK18-BamHI-R	CGGGATCCTTGGAGAATGATACTAAAAA AGATTTGAGTAAGAAA	Dual-LUC
ProMAPKKK18-F3S	CTTTGTTTTCATGCCACGTGTTGTCTCTA CCG	Probe 3 for EMSA
ProMAPKKK18-R3S	CGGTAGAGACAACACGTGGCATGAAAA CAAAG	Probe 3 for EMSA
ProMAPKKK18-F2S	TACCTCTGCCGACACGTGGCTCGAGACA AAT	Probe 2 for EMSA
ProMAPKKK18-R2S	ATTTGTCTCGAGCCACGTGTCGGCAGAG GTA	Probe 2 for EMSA
ProMAPKKK18-F1	CGAAATTGGATCACATTTGGTAATCAAC	Probe 1 for EMSA
ProMAPKKK18-R1	GCGAAAATAAATAAGTGTACGTATGTAA TCG	Probe 1 for EMSA
ProMAPKKK18-mP1-S	CGAAATTGGATCACATTTGGTAATCAACT TTTTTTTCCAAAGTCTCTAATCATATGGAC GATTACATACGTACACTTATT TATTTTCGC	EMSA
ProMAPKKK18-mP1-AS	GCGAAAATAAATAAGTGTACGTATGTAAT CGTCCATATGATTAGAGACTTTGGAAAAA AAAGTTGATTACCAAATGTGAT CCAATTTCG	EMSA
3xABRE-S	GCTGAGACGTGGCAGGACGACGTGGCA GGACACGTGGCGTAA	EMSA
3xABRE-AS	TTACGCCACGTGTCCTGCCACGTCGTCCT GCCACGTCTCAGC	EMSA
3xmABRE-S	GCTGAGAATCAATAGGACGAATCAATAG GACAATCAATGTAA	EMSA
3xmABRE-AS	TTACATTGATTGTCCTATTGATTCGTCCTA TTGATTCTCAGC	EMSA
ProMAPKKK18-ChIP-F3	TGGTAAACAACGGTGAACGA	ChIP-qPCR
ProMAPKKK18-ChIP-R3	CGTGTGAGCGGTAGAGACAA	ChIP-qPCR

ProMAPKKK18-ChIP-F2	AAATTTCCGTGCTTGCACTT	ChIP-qPCR
ProMAPKKK18-ChIP-R2	GAAGGTGATGACGTGTCGAA	ChIP-qPCR
ProMAPKKK18-ChIP-F1	GTTCACTGCCGCGTCACTCC	ChIP-qPCR
ProMAPKKK18-ChIP-R1	AAGGGTTTCAGATTAATTTTGGTTCG	ChIP-qPCR
ProMAPKKK18-ChIP-CK-F	CACCAACCGTCCTGTAATGA	ChIP-qPCR
ProMAPKKK18-ChIP-CK-R	AGAACACAGTGGAGGAAAGGA	ChIP-qPCR
ACT7-ChIP-F	CGTTTCGCTTTCCTTAGTGTTAGCT	ChIP-qPCR
ACT7-ChIP-R	AGCGAACGGATCTAGAGACTCACCTTG	ChIP-qPCR
GABI_244-G02-LP	GAGGAAAAACTTTAGGCCGTG	*mapkkk18* genotyping
GABI_244-G02-LP	TGTGAACCGACTAATTCCGTC	*mapkkk18* genotyping
SALKseq_034842-LP	AATTCGGCACCTTTTTGTTG	*mapkkk18* genotyping
SALKseq_034842-RP	CTTCTTATGGAGTACGCGCC	*mapkkk18* genotyping
SALKseq_087047-F	AAGGGGAAGATGAATTGAGG	*mapkkk18* genotyping
SALKseq_087047-R	GGAGAGATGGACAGCGAGTC	*mapkkk18* genotyping
SALKseq_123341-F	CCACGGCCTAAAGTTTTTCC	*mapkkk18* genotyping
SALKseq_123341-R	TTTTTCCTCATTTGAGAACGTG	*mapkkk18* genotyping
SALK_132819-LP	CCGGTAAGGGTTCTTCTCAAG	*abf1* genotyping
SALK_132819-RP	AGAGGCAACAGACTTTAGGGG	*abf1* genotyping
SALK_002984-LP	TAATGGGAAATCTTGGTGCAG	*abf2* genotyping
SALK_002984-RP	TCTTTTGCATTTCCATGATCC	*abf2* genotyping
SALK_096965_LP	ACACTGTTATTAACGGCGGTG	*abf3* genotyping
SALK_096965_RP	CTTTCTCCAGAACTGCACCTG	*abf3* genotyping
SALK_069523-LP	TCCTCGATTAAGCACATACGG	*abf4* genotyping
SALK_069523-RP	GAACAAGGGTTTTAGGGCTTG	*abf4* genotyping
SALK_061079-LP	TCTTCTTTGATGGGTGGTTTG	*aerb3* genotyping
SALK_061079-RP	CAAGCTTGGCTATTGCAGAAC	*aerb3* genotyping
SALK_013163_LP	CAATGGAAGTTCGGAATCATG	*abi5* genotyping
SALK_013163_RP	CACTCGTTTTCTTCTTAAAGCG	*abi5* genotyping
ABF1-NcoI-F	CGCCATGGGTACTCACATTGATAT	Dual LUC, overexpression, semi-qRT-PCR
ABF1-SalI-R	CGCGTCGACCCACGGACCGGTAAGGGTTC	Dual LUC, overexpression, semi-qRT-PCR
AtABF1-GW-BamHI-F	AAAGCAGGCTTCcATggatccATGGGTACTCACATTGAT	GST fusion for prokaryotic expression
ABF1-CE-XhoI-R	CCCTCGAGCCACGGACCGGTAAGGGTTC	GST fusion for prokaryotic expression

ABF2-BspHI-F	TTAGGTCATGATCGATGGTAGTATGAATTTGGG	Dual LUC, overexpression semi-qRT-PCR
ABF2_SalI_R	CGCGTCGACCCAAGGTCCCGACTCTGTCC	Dual LUC, overexpression, semi-qRT-PCR
ABF2-CE-BamHI-F	CGGGATCCATGGATGGTAGTATGAATTTGGG	GST fusion for prokaryotic expression
ABF2-CE-XhoI-R	CCCTCGAGCCAAGGTCCCGACTCTGTCC	GST fusion for prokaryotic expression
ABF3-BspHI-F	TTAGGTCATGATCGGGTCTAGATTAAACTTCAA	Dual LUC, overexpression, semi-qRT-PCR
ABF3_SalI_R	CGCGTCGACCCAGGGACCCGTCAATGTCC	Dual LUC, overexpression, semi-qRT-PCR
ABF3_CE_BamHI_F	CGGGATCCATGGGGTCTAGATTAAACTTCAAGA	GST fusion for prokaryotic expression
ABF3-CE-XhoI_R	CCCTCGAGCCAGGGACCCGTCAATGTCC	GST fusion for prokaryotic expression
ABF4-BspHI-F	TTAGGTCATGATCGGAACTCACATCAATTTCAA	Dual LUC, overexpression, semi-qRT-PCR
ABF4_SalI_R	CGCGTCGACCCATGGTCCGGTTAATGTCC	Dual LUC, overexpression, semi-qRT-PCR
ABF4_CE_BamHI_F	CGGGATCCATGGGAACTCACATCAATTTCAAC	GST fusion for prokaryotic expression
ABF4-CE-XhoI_R	CCCTCGAGCCATGGTCCGGTTAATGTCCT	GST fusion for prokaryotic expression
AREB3_BamHI_F	CGGGATCCATGGATTCTCAGAGGGGTATT	semi-qRT-PCR
AREB3-XhoI-R	CCCTCGAGGAAAGGAGCCGAGCTTG	semi-qRT-PCR
ABI5_BamHI_F	CGGGATCCATGGTAACTAGAGAAACGAAG	semi-qRT-PCR
ABI5_XhoI_R	CCCTCGAGGAGTGGACAACTCGGGTT	semi-qRT-PCR, Dual LUC
ABI5_BspHI_F	TTAGGTCATGATCGTAACTAGAGAAACGAAGTT	Dual LUC,
RD29B-qPCR_F	CAAAGCGAGAGCCCTGTAAAA	qRT-PCR
RD29B-qPCR_R	CGTAAACCGGAGTCAACTTCTCA	qRT-PCR
RAB18-qPCR-F	TGGACAAGGAGGGAGGAGGAAGAA	qRT-PCR
RAB18-qPCR-R	ACCACCGTAGCCACCAGCATCATA	qRT-PCR
NYE1-qPCR-F	TGGGCAAATAGGCTATACCG	qRT-PCR
NYE1-qPCR-R	CCACCGCTTATGTGACAATG	qRT-PCR
AtNYE2-qPCR-F	GAGGCCATGTCGCAGCACCAG	qRT-PCR
AtNYE2-qPCR-R	ACAGGCCATCCGCAACATTCTCA	qRT-PCR
NYC1-qPCR-F	AGGAGAGGCCGATGGTTTGATG	qRT-PCR
NYC1-qPCR-R	TGTTCTCTGCCCAGTTCCTTAGTC	qRT-PCR
AtPAO-qPCR-F	TCCAAGGTGCGAATGATGACA	qRT-PCR

AtPAO-qPCR-R	TTACGGGCGCTGCAAACGATGGA	qRT-PCR
SAG29-qPCR-F	CTGTTTTCGCTGCCCCTCT	qRT-PCR
SAG29-qPCR-R	ACAGCCCTAGTACGAATCCCAC	qRT-PCR
UBQ10-qPCR-F	GGCCTTGTATAATCCCTGATGAA	qRT-PCR
UBQ10-qPCR-R	AGAAGTTCGACTTGTCATTAGAAAGAAA	qRT-PCR
UBC21-qPCR-F	CTTAACTGCGACTCAGGGAAT	qRT-PCR
UBC21-qPCR-R	GGCGAGGCGTGTATACATTT	qRT-PCR

Notes: F and R represent forward (sense) and reverse (anti-sense) primers, respectively. Restriction sites are underlined.

References:

6. Gao, S., Gao, J., Zhu, X., Song, Y., Li, Z., Ren, G., Zhou, X., and Kuai, B. (2016) ABF2, ABF3, and ABF4 promote ABA-mediated chlorophyll degradation and leaf senescence by transcriptional activation of chlorophyll catabolic genes and Senescence-Associated Genes in Arabidopsis. Mol Plant 9, 1272-1285

53. Yang, L., Ye, C., Zhao, Y., Cheng, X., Wang, Y., Jiang, Y.-Q., and Yang, B. (2018) An oilseed rape WRKY-type transcription factor regulates ROS accumulation and leaf senescence in Nicotiana benthamiana and Arabidopsis through modulating transcription of RbohD and RbohF. Planta 247, 1323-1338

55. Niu, F., Wang, C., Yan, J., Guo, X., Wu, F., Yang, B., Deyholos, M. K., and Jiang, Y. Q. (2016) Functional characterization of NAC55 transcription factor from oilseed rape (Brassica napus L.) as a novel transcriptional activator modulating reactive oxygen species accumulation and cell death. Plant Mol Biol 92, 89-104

58. Clough, S. J., and Bent, A. F. (1998) Floral dip: a simplified method for Agrobacterium-mediated transformation of Arabidopsis thaliana. Plant J 16, 735-743

59. Shu, K., Chen, Q., Wu, Y., Liu, R., Zhang, H., Wang, P., Li, Y., Wang, S., Tang, S., Liu, C., Yang, W., Cao, X., Serino, G., and Xie, Q. (2016) ABI4 mediates antagonistic effects of abscisic acid and gibberellins at transcript and protein levels. Plant J 85, 348-361

60. Zheng, Y., and Perry, S. E. (2011) Chromatin immunoprecipitation to verify or to identify in vivo protein-DNA interactions. Methods Mol Biol 754, 277-291
